# KidneyNetwork: using kidney-derived gene expression data to predict and prioritize novel genes involved in kidney disease

**DOI:** 10.1038/s41431-023-01296-x

**Published:** 2023-02-20

**Authors:** Floranne Boulogne, Laura R. Claus, Henry Wiersma, Roy Oelen, Floor Schukking, Niek de Klein, Shuang Li, Harm-Jan Westra, Bert van der Zwaag, Franka van Reekum, Genomics England Research Consortium, Dana Sierks, Ria Schönauer, Zhigui Li, Emilia K. Bijlsma, Willem Jan W. Bos, Jan Halbritter, Nine V. A. M. Knoers, Whitney Besse, Patrick Deelen, Lude Franke, Albertien M. van Eerde

**Affiliations:** 1 Department of Genetics, University Medical Center Groningen, University of Groningen, Groningen, The Netherlands; 2 Oncode Institute, Utrecht, The Netherlands; 3 Department of Genetics, University Medical Center Utrecht, Utrecht, The Netherlands; 4 Genomics Coordination Center, University of Groningen, University Medical Center Groningen, Groningen, The Netherlands; 5 Department of Nephrology, University Medical Center Utrecht, Utrecht, The Netherlands; 6 Medical Department III - Endocrinology, Nephrology, Rheumatology Department of Internal Medicine, Division of Nephrology, University of Leipzig Medical Center, Leipzig, Germany; 7 Department of Nephrology and Medical Intensive Care, Charité-Universitätsmedizin Berlin, Corporate Member of Freie Universität Berlin and Humboldt-Universität zu Berlin, Berlin, Germany; 8 Department of Internal Medicine (Nephrology), Yale School of Medicine, New Haven, CT USA; 9 Department of Clinical Genetics, Leiden University Medical Center, Leiden, The Netherlands; 10 Department of Internal Medicine, St Antonius Hospital, Nieuwegein, The Netherlands; 11 Department of Internal Medicine, Leiden University Medical Center, Leiden, The Netherlands

**Keywords:** Gene regulatory networks, Gene expression profiling

## Abstract

**Abstract:**

Genetic testing in patients with suspected hereditary kidney disease may not reveal the genetic cause for the disorder as potentially pathogenic variants can reside in genes that are not yet known to be involved in kidney disease. We have developed KidneyNetwork, that utilizes tissue-specific expression to inform candidate gene prioritization specifically for kidney diseases. KidneyNetwork is a novel method constructed by integrating a kidney RNA-sequencing co-expression network of 878 samples with a multi-tissue network of 31,499 samples. It uses expression patterns and established gene-phenotype associations to predict which genes could be related to what (disease) phenotypes in an unbiased manner. We applied KidneyNetwork to rare variants in exome sequencing data from 13 kidney disease patients without a genetic diagnosis to prioritize candidate genes. KidneyNetwork can accurately predict kidney-specific gene functions and (kidney disease) phenotypes for disease-associated genes. The intersection of prioritized genes with genes carrying rare variants in a patient with kidney and liver cysts identified *ALG6* as plausible candidate gene. We strengthen this plausibility by identifying *ALG6* variants in several cystic kidney and liver disease cases without alternative genetic explanation. We present KidneyNetwork, a publicly available kidney-specific co-expression network with optimized gene-phenotype predictions for kidney disease phenotypes. We designed an easy-to-use online interface that allows clinicians and researchers to use gene expression and co-regulation data and gene-phenotype connections to accelerate advances in hereditary kidney disease diagnosis and research.

**Translational statement:**

Genetic testing in patients with suspected hereditary kidney disease may not reveal the genetic cause for the patient’s disorder. Potentially pathogenic variants can reside in genes not yet known to be involved in kidney disease, making it difficult to interpret the relevance of these variants. This reveals a clear need for methods to predict the phenotypic consequences of genetic variation in an unbiased manner. Here we describe KidneyNetwork, a tool that utilizes tissue-specific expression to predict kidney-specific gene functions. Applying KidneyNetwork to a group of undiagnosed cases identified *ALG6* as a candidate gene in cystic kidney and liver disease. In summary, KidneyNetwork can aid the interpretation of genetic variants and can therefore be of value in translational nephrogenetics and help improve the diagnostic yield in kidney disease patients.

## Introduction

Genetic testing in patients with suspected hereditary kidney disease can reveal causative pathogenic variants in kidney-related genes. However, in many cases, a genetic cause cannot yet be detected. Pathogenic variants in known kidney-related genes are detected in approximately 10–30% of genetically tested patients with chronic kidney disease of any cause [1–3]. However, these percentages are likely underestimations of the number of patients with a monogenic cause as variants in genes not yet implicated in kidney disease will go unnoticed. Potentially harmful variants can reside in these genes, which makes it difficult to prioritize and interpret the relevance of these variants. Therefore, in the current era of genomic medicine, one of the main challenges after a negative diagnostic result in known genes is to detect and prioritize new candidate genes with potentially pathogenic variants that can explain the patient’s disease [4].

RNA-sequencing data can be used to predict candidate disease genes [5]. We recently developed GeneNetwork and the GeneNetwork-Assisted Diagnostic Optimization (GADO) method to prioritize new candidate disease genes based on RNA-sequencing data [6]. The idea behind this method is that certain rare disorders can be caused by variants in several genes. While these genes are different, they usually have similar biological functions. When studying gene expression data from a large number of samples, these disease genes usually show strong co-expression [6]. Thus, if there are other genes that are strongly co-expressed with known rare disease genes, it is possible that variants in these other genes can also cause the same disease.

For this kind of tool to work optimally, the co-expression information should be as accurate as possible. For GADO, we built a gene co-expression network based on publicly available RNA-sequencing datasets from many different tissues and used this network to predict which genes might be causing rare diseases. These predictions were trained using the human phenotype ontology (HPO) database [7]. In the HPO database, genes are assigned to phenotypes ‒ called HPO-terms ‒ that are based on gene‒disease annotations and disease symptoms present in the OMIM [8] and Orphanet [9] databases. By integrating the information from the HPO database with the gene co-expression network, we could calculate prediction scores for each gene per HPO term. Together, these scores constitute GeneNetwork. GADO then prioritizes genes by combining an input list of HPO-terms that describe the patient’s phenotype with a list of genes with possible deleterious variants from that patient. The prioritization of the gene list is based on the combined gene prediction scores for the input HPO-terms [6].

Because we observed that GeneNetwork’s prediction performance for kidney-related HPO phenotypes was limited, we sought to improve prediction by developing a kidney-specific network. We did this by using 878 kidney RNA-sequencing samples that we enriched with an existing dataset of 31,499 samples from other tissues [6]. By developing a new prediction algorithm that can weigh the information that is present within both datasets we improved performance for kidney-related pathways. In this paper we present the resulting KidneyNetwork, a co-expression network that can be used to accurately predict gene‒phenotype associations of genes unknown for kidney-related HPO-terms. As proof of principle, we applied KidneyNetwork to exome sequencing data from a group of patients with previously unresolved kidney diseases.

## Methods

To improve the prediction of kidney-related phenotypes, we collected kidney-derived RNA-sequencing data, updated GeneNetwork with more recent reference databases and improved statistical analyses, followed by integration of tissue-specific information.

### Datasets in KidneyNetwork

RNA-sequencing data from selected kidney samples of several origins, including primary, tumor and fetal tissue were combined with an existing dataset of multi-tissue RNA-sequencing used as the foundation for our previously described GeneNetwork [6] (Table S1, S2). We chose to include the multi-tissue dataset for two reasons. First, we needed a sufficient number of samples to build a baseline network. Second, we wanted to preserve expression that is specific to several, or all, kidney cell types but not to other tissues. We did this because gene‒phenotype scores are based on differences in expression between samples; if all genes have high (or low) expression in all the samples included in the analysis, they will not add sufficient information to the prediction algorithm. The multi-tissue dataset of human RNA-sequencing samples used to develop GeneNetwork was re-used and processed as described previously [6]. After pre-processing, this dataset contained 31,499 samples and 56,435 genes.

3,194 Kidney-derived RNA-sequenced samples were downloaded from the European Nucleotide Archive (ENA) and the Genotype-Tissue Expression (GTEx) Project (Note S1). Preprocessing of the kidney dataset was done similarly to the multi-tissue dataset [6] (Note S2, Note S3). After sample and gene selection, 58,283 genes and 878 kidney samples remained. We investigated the remaining 878 RNA-sequencing samples using the UMAP clustering algorithm (Note S4).

#### HPO filtering

For the construction of KidneyNetwork we used gene‒phenotype associations from HPO database [7] version 1268. In the HPO database, annotation of genes to HPO-defined phenotypes is based on the gene‒disease annotations in the OMIM [8] morbid map (downloaded March 26, 2018) and the Orphanet [9] “en_product6.xml” file version 1.3.1. Gene‒disease annotations in these databases can be based on several factors, including statistical associations and large-scale copy number variations. We wanted to train KidneyNetwork using only genes for which the link between gene and the rare disease is well established. Therefore, we excluded the multigenic syndromes, since it is often not clear which of the genes in the copy number variants contribute to which phenotypes. We also excluded mere susceptibility genes (Note S5).

#### Expression normalization

After sample and gene quality control (QC), the expression matrix of the remaining samples and genes was log_2_-transformed and gene counts were normalized using DESeq following the median of ratios method. We then corrected the gene expression data for covariates (Note S6).

### Decomposition

After filtering and QC of the entire dataset, the next step was to perform a decomposition to calculate the eigenvectors of the dataset (Note S7). For both GeneNetwork and the gene regulatory network based on kidney-derived data, we defined the optimal number of components (Note S8). The first 165 eigenvectors for GeneNetwork and the first 170 eigenvectors for the kidney-derived data were identified and merged into a larger matrix containing all 335 eigenvectors.

### Gene‒HPO-term score calculation

The gene‒phenotype score calculation was done in several steps (Fig. S5). First, we performed a logistic regression using the combined eigenvectors and the gene‒phenotype annotations file as input. We used the resulting β values and the eigenvector scores to calculate a gene log-odds score for every gene in every eigenvector (Note S9).

$$gene_{log - odds - score} = \beta _0 + \beta _1 \cdot eigenvector_1 + \cdots + \beta _n \cdot eigenvector_n$$

To avoid overfitting of the gene log-odds-scores of already annotated genes, we applied a leave-one-out cross validation approach (Note S10). The log-odds were subsequently translated to gene *z*-scores using a permuted null distribution for each phenotype (Note S11).

To determine prediction accuracy, we calculated the area under the ROC-curve (AUC). The AUC was calculated per HPO-term using the predicted gene *z*-scores and known annotations. The significance of the predictions was calculated using the two-sided Mann-Whitney rank test. After Bonferroni-correction, a prediction was considered significant at *p* < 0.05.

### Comparison of prediction performance

We compared the prediction performance of four distinct networks: (1) the original GeneNetwork, (2) the updated GeneNetwork, (3) the kidney-specific gene regulatory network based solely on kidney-derived samples and finally (4) KidneyNetwork, that combines the latter two. The quality of the HPO predictions made by these networks was assessed based on the AUC for each kidney-related phenotype (Table S3). Improved quality of a network was defined as improved prediction accuracy for kidney-related terms that were significantly predicted in each comparison of two networks and by an increased number of significantly predicted kidney-related terms. The significance of improvement in prediction accuracy of one network versus another was assessed using the DeLong test [10] integrated in the pROC R package [11].

### Application of KidneyNetwork to 13 patients with suspected hereditary kidney disease

One of the applications of KidneyNetwork is to prioritize candidate genes in patients with unsolved kidney disease. To evaluate this clinical application, we used KidneyNetwork to prioritize candidate genes for patients with various kidney diseases using the GADO method [6]. GADO combines the gene prediction *z*-scores rendered through KidneyNetwork for a given set of HPO-terms. Genes with a combined *z*-score ≥5 for the unique set of HPO-terms associated with each patient were considered potential candidate genes for that patient.

The 13 patients included in the study were all suspected to have a monogenic kidney disease, but had no genetic diagnosis (Note S12). HPO-terms were assigned to these cases based on their phenotype. For each patient, the complete exome sequencing data were analyzed using CAPICE [12] to identify potentially pathogenic variants. Genes containing variants with a gnomAD Popmax filtering AF [13] <0.005 and a recall ≥99%, corresponding with a mild CAPICE cut-off of ≥0.0027, were considered interesting candidates.

Overlapping the genes identified by the KidneyNetwork integration in GADO with those identified by CAPICE resulted in a list of genes for each patient. These genes and variants in these genes were manually reviewed by a nephrogenetics expert panel (AMvE, LRC, NVAMK) for their pathogenetic potential based on population metrics, prediction tools, available literature and segregation (Note S13). For the resulting candidate gene, additional patients carrying variants in the same gene were identified via collaborators and the 100,000 Genomes Project [14]. Also the GeneMatcher tool [15] was used, and yielded no additional patients through February 15th, 2023.

### Identification of additional patients

The previously described unsolved polycystic kidney and liver disease cohort [16] was used to assess rare variants (Note S14). We used a Fisher’s exact test to compare the frequency of identified variant(s) to the European subset of non-Finnish Europeans in the gnomAD database [17]. Furthermore, we used the 100,000 Genomes Project [14] for identification of additional patients based on the identified variant(s) (Note S15).

## Results

### Data retrieval and sample clustering

**Fig. 1:**
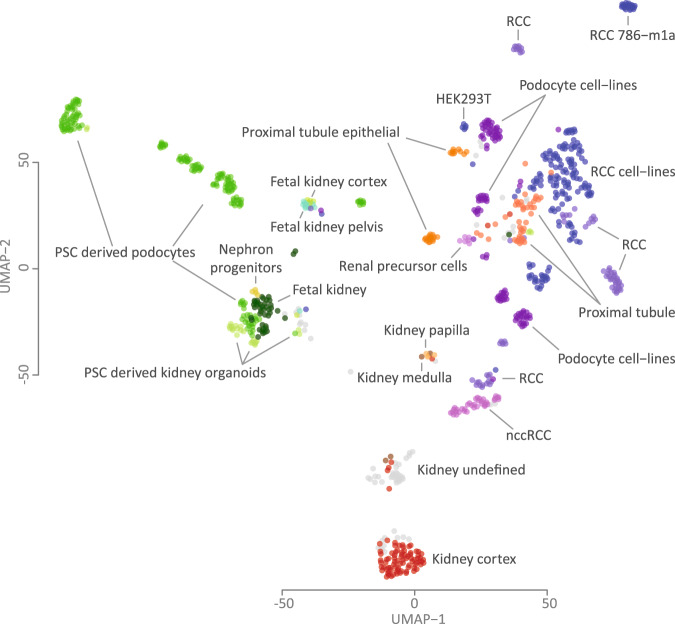
UMAP visualization of the kidney-derived expression data. 878 samples group into three main clusters: healthy primary tissue (middle and bottom), developmental samples (left) and renal cell carcinoma (RCC) samples (right). On the left side of the figure, clustering of pluripotent stem cell (PSC)-derived podocytes and PSC-derived organoids with primary fetal samples and nephron progenitor cells can be seen. On the right side, RCC samples cluster close to proximal tubule samples, and the RCC cluster closest to healthy primary tissue samples consists of non-clear cell RCC (nccRCC) samples. In the middle and at the bottom, healthy primary kidney samples cluster based on their tissue of origin.

We selected 878 kidney samples (Fig. S2), which we clustered and plotted using the UMAP algorithm (Fig. 1). Generally, the data clusters into three main clusters: primary non-tumor kidney data, kidney developmental samples and proximal tubule, glomerulus and renal cell carcinoma (RCC) samples.

### KidneyNetwork improves gene‒phenotype predictions

**Fig. 2:**
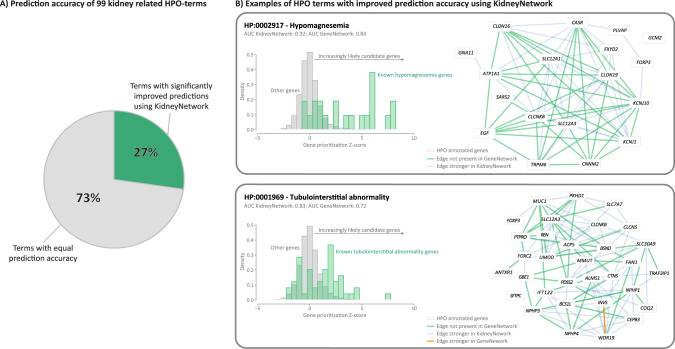
KidneyNetwork performs better for kidney-related HPO-terms than the updated GeneNetwork. **A** 27% of kidney-related phenotypes are predicted significantly better using KidneyNetwork, as compared to GeneNetwork. **B** Density plots of the gene prediction scores within two of the most improved phenotypes, hypomagnesemia and tubulointerstitial abnormality, show higher prediction values for the genes annotated for the phenotype and also predict potential unknown candidate genes. The networks predicted using KidneyNetwork shows more and stronger correlations between the annotated genes than the networks predicted using GeneNetwork.

First, we updated GeneNetwork with the updated HPO database (Fig. S6) and optimized the gene network building pipeline (Fig. S7). These changes yielded an improvement in the general GeneNetwork compared to the previous version (Fig. S8). We then used the improved pipeline to build the kidney-specific gene regulatory network. As expected, given the small sample size, this version of the kidney-specific network performed less well than GeneNetwork (Fig. S9). Subsequently, combining GeneNetwork and the kidney specific gene co-expression network into KidneyNetwork yielded our best results for kidney-related HPO-terms (Fig. 2A; Table S5). The prediction AUC, precision, sensitivity and f1-scores for each predicted pathway are provided (Table S6).

We calculated the number of pathways with a significant improvement in prediction accuracy for KidneyNetwork compared to GeneNetwork using the DeLong test [10]. For this analysis, phenotypes were grouped into kidney-related phenotypes and non-kidney-related phenotypes. Within the kidney-related phenotypes, no phenotypes were significantly better predicted in GeneNetwork compared to KidneyNetwork. In contrast, 27% of kidney-related pathways were significantly better predicted by KidneyNetwork compared to GeneNetwork (Fig. 2A). For these pathways, a mean AUC increase from 0.73 to 0.81 was observed (*t*-test *p*-value: 1.813e-10). This indicates that, overall, kidney-related terms can be predicted with a higher accuracy using KidneyNetwork compared to GeneNetwork.

Two examples of improved kidney-related HPO-terms are hypomagnesemia and tubulointerstitial abnormality (Fig. 2B). Visualization of these phenotypes in density plots shows higher prioritization *z*-scores for known disease-related genes compared to non-annotated genes. For unknown genes, the higher the prediction *z*-score, the more likely they are to be a candidate disease gene. Visualizing the gene interaction networks of known disease genes based on the prediction scores again shows the increase in the number and strength of interactions obtained using KidneyNetwork compared to GeneNetwork.

We also saw an increase in the number of significant predicted kidney-related HPO-terms for KidneyNetwork (*n* = 71) compared to GeneNetwork (*n* = 63). This led us to hypothesize that KidneyNetwork predicts kidney-related terms with higher accuracy overall and is therefore capable of predicting more kidney-related phenotypes with higher significance. A paired *t*-test shows that overall, the HPO AUC score was significantly better for KidneyNetwork versus GeneNetwork (mean AUC: 0.76 versus 0.74; *t*-test *p*-value: 4.5 × 10^−8^). This result suggests that KidneyNetwork predicts more kidney-specific HPO-terms with a higher prediction accuracy than GeneNetwork.

### KidneyNetwork prioritizes *ALG6* as candidate disease gene in patient with kidney cysts and liver cysts

**Fig. 3:**
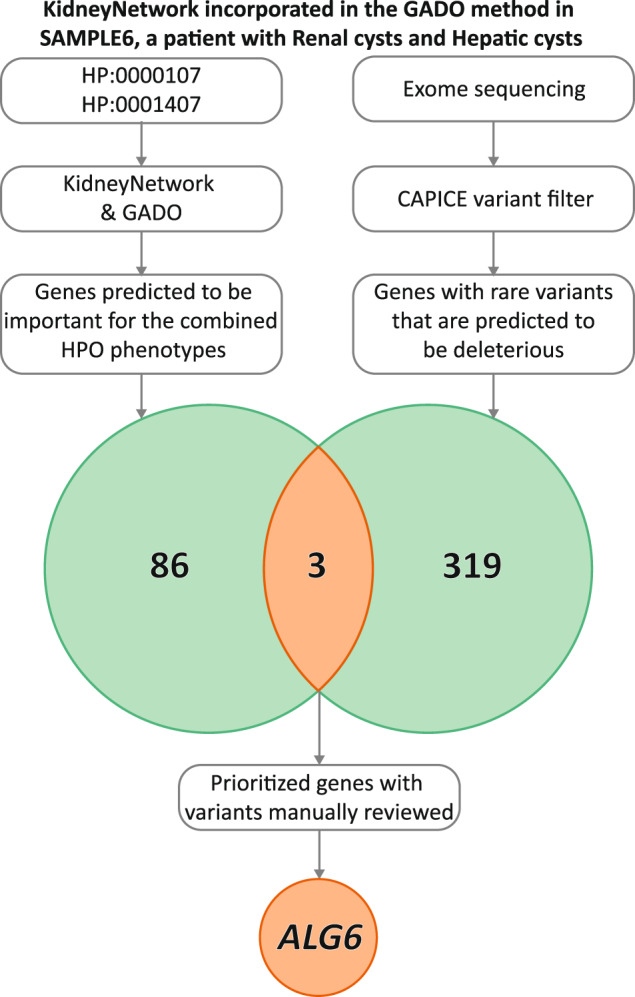
KidneyNetwork incorporated in the GADO method in SAMPLE6, a patient with renal and hepatic cysts. 89 candidate genes out of all genes were prioritized by KidneyNetwork using GADO, based on the HPO-terms “Renal cysts” (HP:0000107) and “Hepatic cysts” (HP:0001407). Exome-sequencing data interpretation method CAPICE yielded 322 genes containing potentially pathogenic variants in the patient’s exome sequencing data. When overlapping these gene lists three genes were identified that met the selection criteria, one being *ALG6*.

To examine the clinical utility of KidneyNetwork, we prioritized genes for 13 patients with a suspected hereditary kidney disease but no genetic diagnosis and intersected these with genes containing potentially pathogenic variants. The resulting gene lists contained 1‒4 candidate genes for 9 of the 13 patients (Table S7). In one patient (SAMPLE6), manual curation of this list identified *ALG6* (*ALG6* alpha-1,3-glucosyltransferase) as a potential candidate gene to explain the patient’s kidney and liver cysts (Fig. 3). The combined *z*-score for *ALG6* for the imputed HPO-terms was significant in KidneyNetwork after multiple testing correction (*z* = 5.43). This gene would have been missed if we had used GeneNetwork: there *ALG6* did not reach the significance threshold of *z*-score ≥5.

### *ALG6* as candidate gene for patients with kidney and liver cysts

The *ALG6* variant c.680 + 2 T > G carried by SAMPLE6 is heterozygous. This is a known pathogenic splice site variant that results in congenital disorder of glycosylation (CDG) type Ic when pathogenic variants are present on both alleles [18, 19]. *ALG6* strongly resembles *ALG8* which has been implicated in kidney and liver cyst phenotypes [20], and according to KidneyNetwork, *ALG6* and *ALG8* are highly co-regulated (*z*-score = 8.59).

Given this biological plausibility, we queried a cohort of 120 unrelated cases of polycystic kidney and liver disease for rare variants, MAF < 0.001, in *ALG6*. This cohort is minorly updated since it was previously described and has been excluded by exome sequencing analysis for loss of function mutations or reported pathogenic non-truncating variants in *PKD1, PKD2, PRKCSH, SEC63, GANAB, ALG8, ALG9, SEC61B, PKHD1*, or *DNAJB11* [16]. Three unrelated cases (YU372, YU378, YU481) carried rare *ALG6* variants; each had the same *ALG6* c.257 + 5 G > A non-canonical splice variant known to be pathogenic for *ALG6*-CDG and splice-altering in vitro [19, 21]. Despite a shared mutation, these three cases each report no known affected family members, were enrolled from different states across the United States, and are unrelated to the best of limit of detection using VCFtools relatedness2 algorithm with Relatedness_PHI < 0.005.

Given the representation of this variant in three cases of European ancestry in this phenotypically-defined cohort, we compared its frequency in the European subset of cases (*n* = 105) to non-Finnish Europeans in gnomAD [17] with coverage at this position (*n* = 64,466) [17]. In the patient cohort 3 out of 210 alleles contained this variant, while in gnomAD, a cohort unselected with regards to kidney or liver cyst burden, it was found in 121 of 128,932 alleles. This approximately 10-fold enrichment is statistically significant by Fishers exact test, *p* = 0.0011. This mutation was also recurrent in cases of *ALG6*-CDG [19].

We also investigated the 100,000 Genomes Project dataset [14] and contacted collaborators which identified three additional patients with kidney and/or liver cysts carrying a heterozygous potentially deleterious variant in *ALG6*, without an alternative genetic explanation.

**Fig. 4:**
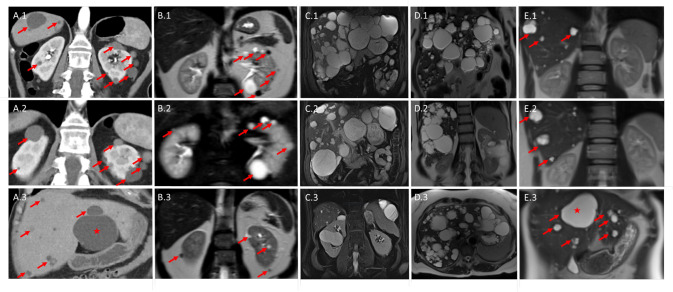
Imaging from patients. **A** abdominal CT illustrating polycystic kidneys and liver in SAMPLE6. Some cysts are highlighted by red arrows, with the largest hepatic cyst measuring 7.7 cm (red asterisk). **B** abdominal MRI of affected child of SAMPLE6 shows multiple cysts in left kidney (several highlighted with red arrows), some hypo-intense on T2 and few cysts in right kidney. **C** abdominal MRI of YU378 showing extensive polycystic liver disease and two kidney cysts. **D** abdominal MRI of YU481 shows multiple liver cysts. Left kidney has 9 cm cyst and a few small cysts, right kidney no cysts. **E** abdominal MRI illustrating polycystic liver disease in LE1. Hepatic cysts are highlighted by red arrows, with the largest cyst located in liver segment IV (red asterisk), necessitating surgical intervention for progressive cholestasis. Of note, both kidneys presented with normal morphology in absence of any cystic lesions.

In total, we identified seven patients with known splice site variants that were reported to be disease-causing in severely affected CDG patients upon homozygosity or compound-heterozygosity and one patient with a likely pathogenic splice site variant (Table 1). In contrast to the severely affected *ALG6*-CDG patients (presenting with multi-organ involvement including developmental delay and multiple neurological symptoms), our patients presented with a phenotype of multiple kidney cysts and/or liver cysts (Fig. 4). While PCLD can be extensive, the kidney phenotype seems to be mild with no eGFR decline reported despite advanced age (i.e. one patient is in her thirties, the others are between 45 to 80 years old). Furthermore, we found that the *ALG6* variant segregated in a few family members that were also affected (Table 1; Fig. 4).

**Table 1:** Clinical information on patients with heterozygous *ALG6* variants, including variant details and in silico predictions.

Patient (gender)	Phenotype	Family history and segregation	Variant nomen (cDNA)^1^	Variant nomen (protein)	Zygosity	Allele frequency gnomAD v2.1.1	CADD score (PHRED) v1.5	Reference for variant
SAMPLE6 (female)	multiple renal cysts and multiple hepatic cysts (incidental finding), normal eGFR	child and sibling with renal cysts carry same heterozygous variant. Sibling has cyst complex with septation in right kidney^2^	c.680 + 2 T > G	splice site variant	heterozygous	8.02e-6	29.3	Morava et al., Sun et al.^18,19^
YU372 (female)	symptomatic polycystic liver disease diagnosed at age 51, two kidney cysts	no affected family members known	c.257 + 5 G > A	splice site variant	heterozygous	4.72e-4	22.0	Imbach et al., Westphal et al., Drijvers et al.^21,27,28^
YU378 (male)	symptomatic polycystic liver disease, diagnosed at age 71, small number of kidney cysts, some large	no affected family members known	c.257 + 5 G > A	splice site variant	heterozygous	4.72e-4	22.0	Imbach et al., Westphal et al., Drijvers et al.^21,27,28^
YU481 (female)	symptomatic polycystic liver disease at age 68, left kidney 9 cm cyst and a few small cysts, no cysts in right kidney	no affected family members known	c.257 + 5 G > A	splice site variant	heterozygous	4.72e-4	22.0	Imbach et al., Westphal et al., Drijvers et al.^21,27,28^
GEL2 (male)	multiple renal cysts	no affected family members known	c.257 + 2dup	splice site variant	heterozygous	1.59e-5	25.2	Newell et al.^29^
LE1 (female)	mild polycystic liver disease without renal manifestation, normal eGFR	sibling with liver cysts (and no kidney cysts) carries same heterozygous variant. Unaffected child has biallelic *ALG6* wildtype	c.257 + 5 G > A	splice site variant^3^	heterozygous	4.72e-4	22.0	Imbach et al., Westphal et al., Drijvers et al.^21,27,28^
LU1 (female)	multiple renal cysts, normal eGFR	monozygotic twin affected	c.257 + 5 G > A	splice site variant	heterozygous	4.72e-4	22.0	Imbach et al., Westphal et al., Drijvers et al.^21,27,28^
AN1 (male)	multiple renal cysts, repeated kidney stones, normal eGFR	sibling with kidney stones, no known cysts	c.82_82 + 8del	splice site variant^4^	heterozygous	3.19e-5	24.0	ClinVar 558193

*eGFR* estimated glomerular filtration rate, *NA* not applicable.

^1^NM_013339.4, genome build GRCh37.

^2^Referred to the urologist for further characterization.

^3^Splice site analysis presented in Supplementary Fig. 10.

^4^Likely pathogenic variant, predicted to affect splice site, but this prediction has not been confirmed by published transcriptional studies.

## Discussion

We present KidneyNetwork, a publicly available co-expression network with optimized expression and phenotype annotation data for application to kidney diseases. A significant proportion of patients with a suspected genetic kidney disease remain without a genetic diagnosis, as lists of disease genes for many conditions are incomplete. Identifying which genes are involved in kidney disease is essential for improving the diagnostic yield in kidney disease patients and for studying disease pathogenesis to approach treatment avenues. Establishing novel disease genes requires careful biological validation. Implicating genes worthy of such investigations is critical. Application of KidneyNetwork in conjunction with WES or GWAS data by nephrologists, clinical geneticists, or researchers will help each of these groups to participate in gene implication. KidneyNetwork combines a co-expression network based on a kidney sample dataset with the previously published multi-tissue dataset used to build GeneNetwork. Combining the datasets into KidneyNetwork improved phenotype predictions related to kidney disease, when compared to networks based on the two datasets separately. As proof of principle, we show that the candidate gene list for the combined phenotype of kidney and liver cysts generated by KidneyNetwork prioritized a manageable list of candidate genes from a long list of genes containing rare variants in our patient with this phenotype.

Our implication and exploration of *ALG6* as a potential candidate gene for kidney and liver cysts results in a plausible candidate gene, supported by co-occurrence of the *ALG6* loss of function variants and polycystic liver and kidney disease in several patients with supportive familial segregation of affected patients in two families, and by the statistically significant enrichment of the truncating *ALG6* c.257 + 5 G > A variant in a phenotypically defined cohort of unsolved ADPKD/PCLD cases. Biological validation will be necessary to finally determine if *ALG6* is a disease gene for autosomal dominant polycystic kidney and liver phenotypes.

The biological plausibility is suggested by known functional similarities and tight transcriptional coregulation of *ALG6* to established disease genes as highlighted by KidneyNetwork. *ALG6*, similarly to established polycystic kidney and liver disease gene *ALG8*, is a member of the α3-glucosyltransferase family [22]. In addition to *ALG8* [20], *ALG9* heterozygous variants have recently also been implicated in the etiology of kidney and liver cyst phenotypes [16]. These three genes each play an essential role in the biosynthetic pathway for lipid-linked oligosaccharides prior to their transfer onto asparagine (N) residues of nascent proteins as so-called N-glycans in the endoplasmic reticulum [23]. Interestingly, while kidney or liver cysts have been described, among multi-organ involvement in fetuses or children with *ALG9*-CDG or infrequently in *ALG8*-CDG, cysts have not been described for *ALG6*-CDG [19]. Parents of CDG patients have not yet been studies for cysts. Given the mild phenotype, cysts are likely to go unnoticed in many cases, especially in early parenthood, which is when children are most often diagnosed with CDG.

The phenotype in the genetically unsolved polycystic kidney and liver patients we identified to carry *ALG6* variants is relatively mild, in many cases liver predominant and asymptomatic, consistent with the phenotype described for patients carrying a heterozygous *ALG8* or *ALG9* pathogenic variant. The potentially pathogenic variants we identified are also found in individuals in the gnomAD database [17]. One explanation for this observation could be incomplete penetrance of the disease. The fact that some of the individuals in our cohort reported no known affected family members, could be an indication of incomplete penetrance, although segregation is lacking in many families. However, we did not identify unaffected individuals carrying the variant. An alternative explanation could be that the observed phenotype is relatively mild and subclinical. For example, the kidney and liver cysts observed in SAMPLE6 were discovered as incidental finding. If no abdominal imaging is done in individuals carrying these variants, the cysts can go unnoticed. Also for *ALG8* and *ALG9* Besse et al. contemplate on the relatively mild phenotype and propose this can likely be determined by two factors [16, 20]. First, it is expected that a somatic second hit is needed to get a cystic phenotype. The relative infrequency of these somatic second hit mutations that inactivate the normal copy of *ALG8/ALG9* and the incomplete effect this has on Polycystin-1 is expected to cause a relatively mild phenotype.

*ALG6* has previously been suggested to be involved in one individual with ADPKD [24]. However, that patient, who carried two missense variants with inconclusive predictions that have not been functionally assessed, had a very severe phenotype that did not match the expected phenotype for *ALG6*.

### Strengths and limitations

Building gene co-expression networks requires a large number of RNA-sequencing samples [6] derived from various cell-types and developmental stages in order to achieve accurate function predictions. This sample diversity, combined with high numbers of samples are not often available for specific tissues. To overcome this issue, one earlier approach used hierarchical similarities between tissue types [25]. However, this solution requires a priori gene selection due to its computational burden. In contrast, our method can be used to make unbiased genome-wide predictions. Moreover, the hierarchical approach would have to be repeated for each new tissue of interest, whereas the multi-tissue dataset can be re-used to build a different tissue-specific network using our method. Another approach used differential expression between different tissue types [26]. Here, the top 10% most differentially expressed genes were correlated with kidney-related GWAS loci. Using differential expression allows predictions to be made regardless of previous knowledge on gene‒phenotype interactions. However, this also requires applying a differential expression cut-off. In contrast, our approach makes use of underlying biological structures in RNA-sequencing data to obtain a prediction score for every gene. While combining differential expression with GWAS summary statistics allows for unbiased gene predictions, the reliability of experimentally validated HPO annotations is higher than that of GWAS results. Integrating the HPO database thus results in more reliable predictions. Moreover, we make simultaneous predictions for all HPO-terms, whereas the GWAS-based approach needs to be repeated for each GWAS of interest.

Combining kidney-specific RNA-sequencing samples with the multi-tissue dataset allowed us to overcome both the issue of sample size and the challenges in observing tissue-specific differential expression when using only tissue-specific expression datasets. In addition, during the development of KidneyNetwork we did not have to limit the number of genes that the network is built upon. Furthermore, KidneyNetwork users can get predictions for all possible genes in an unbiased approach, and gene prioritizations for a combination of HPO-terms can be obtained.

A downside of using bulk RNA-sequencing data is that we have limited power to make inferences for lowly expressed genes, which is particularly important for genes that are specific to rare cell-types. As more cell-type specific and single-cell RNA-sequencing data becomes available in the future, creating co-expression networks based on different kidney cell-types might solve this for genes that are expressed more abundantly within specific cell types. Another limitation of using only RNA-sequencing data is that other biological processes potentially involved in disease development, for example post-translational modifications and protein-protein interactions, are currently not considered by our prediction model.

Apart from identifying new plausible candidate genes, KidneyNetwork can also be well used to prioritize known kidney disease genes. This can be particularly useful after an initial negative diagnostic result after exome-based gene panel analysis is performed, which might not include analysis of all known kidney disease genes.

Currently, KidneyNetwork is optimized for intrinsic kidney disease. However, kidney disease can also present because of a pathogenetic process in other systems, such as the immune system. While we can also make inferences on gene prioritization for non-kidney phenotypes, these predictions can improve by building networks specific for different tissues in the future.

We realize that based on the present literature alone, *ALG6* would be a candidate gene for the cyst phenotype in SAMPLE6. To prove involvement of *ALG6* in this phenotype, functional follow-up is required. However, this also proves the strength of our method; out of 322 genes with potentially deleterious variants this plausible candidate gene was prioritized to the top 3, making going into exome-wide sequencing data ‒ for more patients, with various phenotypes ‒ time-efficient and worthwhile.

### Improved gene function predictions

We show that our improved method for assigning gene functions and kidney-related HPO-terms to genes outperforms our previously published model. Our leave-one-out cross validation approach ensures that predictions are not overfitted, that the reported AUC values are not inflated and that our method is robust.

Furthermore, before predicting gene‒phenotypes associations, we excluded gene‒disease associations from the HPO database that had little experimental evidence, because prediction accuracy is dependent on the accuracy of annotated gene‒phenotype associations. Prediction accuracy is based on true positive and true negative gene predictions, which means that more accurately mapping of known genes to phenotypes results in better predictions. Gene-phenotype association accuracy will improve once more genes are annotated and validated for each phenotype. Therefore, we expect an improvement in network prediction accuracy as gene‒phenotype association knowledge increases and is added to the HPO database.

### Applications of KidneyNetwork

We have developed https://kidney.genenetwork.nl/ through which we provide the gene-HPO term prediction. Using the same prediction algorithm that we used to assign genes to HPO-terms, we also predicted which genes are likely to be involved in GO, KEGG and Reactome pathways. Here we also provide an online version of GADO that can be used to prioritize relevant genes for patients with a suspected rare kidney disease. It is possible to specify the phenotype of a patient using HPO-terms and provide a list of genes harboring potential disease-causing variants. These genes will then be ranked using KidneyNetwork, thereby allowing the identification of genes that are more likely to be involved in the patient’s disease. Since it is not necessary to upload personal genetic information, this method respects patient privacy. We advise to use the KidneyNetwork scores in conjunction with WES or GWAS data to increase the prediction accuracy.

### Future directions

Application of KidneyNetwork to unsolved cases from diagnostics, large research cohorts and, for instance, GWAS datasets will result in more insight into kidney physiology and pathophysiology. To further improve the accuracy of kidney phenotype prediction, we plan to build cell-type specific networks by incorporating single-cell RNA-sequencing data, which we expect will yield more detailed and accurate gene‒phenotype predictions.

### Conclusion

We present KidneyNetwork, a kidney-specific co-expression network that accurately predicts which genes have kidney-specific functions. The method we developed to combine multi-tissue data with tissue-specific data can easily be extended to other tissues, allowing improved predictions for other tissue-specific diseases. Using KidneyNetwork, we highlight *ALG6* as candidate gene for kidney and/or liver cysts. KidneyNetwork provides a useful tool to help with the interpretation of genetic variants. It can therefore be of great value in translational nephrogenetics and ultimately improve the diagnostic yield in kidney disease patients.

### Supplementary information

Supplementary tables

Supplementary figures and notes

KidneyNetwork website tutorial

## Data Availability

The publicly available datasets analyzed during the current study are available in the European Nucleotide Archive (ENA) repository (https://www.ebi.ac.uk/ena/browser/home). The GTEx derived datasets are available from the database of Genotypes and Phenotypes (dbGaP), but restrictions apply to the availability of these data, which were used under license for the current study, and so are not publicly available. Data are however available from dbGaP under accession number phs000424.v8.p2. The patient-derived WES datasets analyzed during the current study are not publicly available for privacy reasons. The results are available on kidney.genenetwork.nl.
