# Supplementary material for: KidneyNetwork: using kidney-derived gene expression data to predict and prioritize novel genes involved in kidney disease
Source: Eur J Hum Genet. 2023 Feb 20;31(11):1300–8. doi: 10.1038/s41431-023-01296-x (PMC10620423; doi:10.1038/s41431-023-01296-x)
Supplement: Supplementary file 2 — Supplementary figures and notes [file 41431_2023_1296_MOESM2_ESM.docx]

# **KidneyNetwork: Using kidney-derived gene expression data to predict and prioritize novel genes involved in kidney disease**

# Floranne Boulogne, Laura R. Claus, Henry Wiersma, Roy Oelen, Floor Schukking, Niek de Klein, Shuang Li, Harm-Jan Westra, Bert van der Zwaag, Franka van Reekum, Genomics England Research Consortium, Dana Sierks, Ria Schönauer, Zhigui Li, Emilia K. Bijlsma, Willem-Jan W. Bos, Jan Halbritter, Nine V.A.M. Knoers, Whitney Besse, Patrick Deelen, Lude Franke, Albertien M. van Eerde

# **Supplemental material**

Table of Contents

[Supplementary figures 4](#_Toc110866063)

[Figure S1: Principal component analysis (PCA) was used for sample quality control and selection. 4](#_Toc110866064)

[Figure S2: Flowchart visualizing the sample selection of kidney-derived RNA sequencing data. 5](#_Toc110866065)

[Figure S3: Selecting eigenvector cut-off value for GeneNetwork data based on highest average AUC value of different database. 6](#_Toc110866066)

[Figure S4: Selecting eigenvector cut-off value for kidney-derived samples data based on highest average AUC of different databases. 7](#_Toc110866067)

[Figure S5: Pipeline of the updated network analysis. 8](#_Toc110866068)

[Figure S6: AUC value comparison between the original GeneNetwork and GeneNetwork based on the updated HPO database. 9](#_Toc110866069)

[Figure S7: AUC value comparison between the original GeneNetwork pipeline and the updated GeneNetwork pipeline with the original HPO data. 10](#_Toc110866070)

[Figure S8: AUC value comparison between the original GeneNetwork and the updated GeneNetwork. 11](#_Toc110866071)

[Figure S9: AUC value comparison of kidney-related HPO-terms between GeneNetwork and the network created with kidney-derived data. 12](#_Toc110866072)

[Figure S10: ALG6-splice site analysis c.257+5G>A. 13](#_Toc110866073)

[Supplementary tables 14](#_Toc110866074)

[Table S1. Keywords for kidney sample selection 14](#_Toc110866075)

[Table S2. Sample annotations 14](#_Toc110866076)

[Table S3. Kidney-specific HPO-terms 14](#_Toc110866077)

[Table S4. Diagnostic kidney gene panel (n=379) of the University Medical Centre Utrecht NEF00v18.1 14](#_Toc110866078)

[Table S5. Improved kidney-specific HPO-terms 15](#_Toc110866079)

[Table S6. Phenotypes and genes in 13 kidney disease patients 15](#_Toc110866080)

[Supplementary notes 16](#_Toc110866081)

[Note S1. Kidney-specific RNA-sequencing samples 16](#_Toc110866082)

[Note S2. Kidney-specific RNA-sequencing alignment 16](#_Toc110866083)

[Note S3. Kidney specific sample and gene selection 16](#_Toc110866084)

[Note S4. Sample clustering and investigation using UMAP 17](#_Toc110866085)

[Note S5. HPO filtering 17](#_Toc110866086)

[Note S6. Covariate identification 18](#_Toc110866087)

[Note S7. Decomposition 18](#_Toc110866088)

[Note S8. PC threshold determination 18](#_Toc110866089)

[Note S9. Model fitting 19](#_Toc110866090)

[Note S10. Leave-one-out cross validation 19](#_Toc110866091)

[Note S11. Log-odds to z-score translation 20](#_Toc110866092)

[Note S12. Patient selection and phenotype 20](#_Toc110866093)

[Note S13. (Manual) assessment of potential candidate genes 21](#_Toc110866094)

[Note S14. Unsolved polycystic kidney and liver disease cohort 21](#_Toc110866095)

[Note S15. 100,000 genomes project 21](#_Toc110866096)

## **Supplementary figures**

Figure S1: Principal component analysis (PCA) was used for sample quality control and selection. A cut-off of 0.030 was chosen, and all samples above the threshold were considered good quality. Right figure is based on the same data as the left figure but has an expanded x-axis to produce a more detailed plot.

| 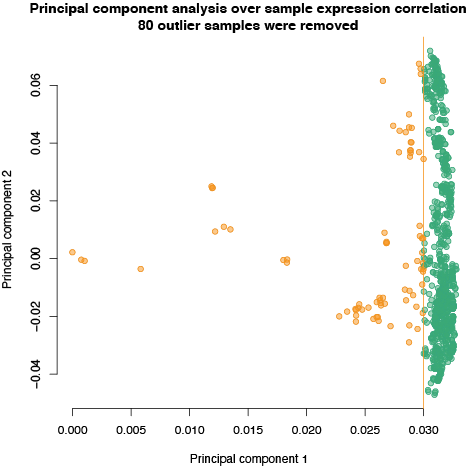 | 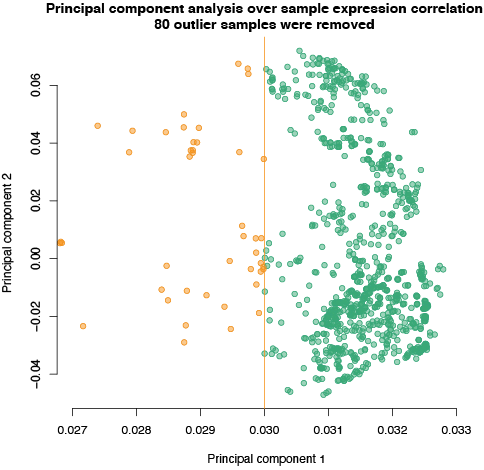 |
| --- | --- |

Figure S2: Flowchart visualizing the sample selection of kidney-derived RNA sequencing data. RNA-seq = RNA-sequencing, ENA = European Nucleotide Archive, dbGaP = database of Genotypes and Phenotypes, PCA = principal component analysis, ssRNA-seq = strand-specific RNA sequencing

Figure S3: Selecting eigenvector cut-off value for GeneNetwork data based on highest average AUC value of different database. An explained variance cut-off of 0.5 gives the highest prediction accuracy for GeneNetwork, averaged over several databases. Each colored line represents the mean AUC of the prediction of all pathways within one database. Black line represents the average AUC over all the databases included. The number of eigenvectors included was based on the explained variance cut-off chosen.


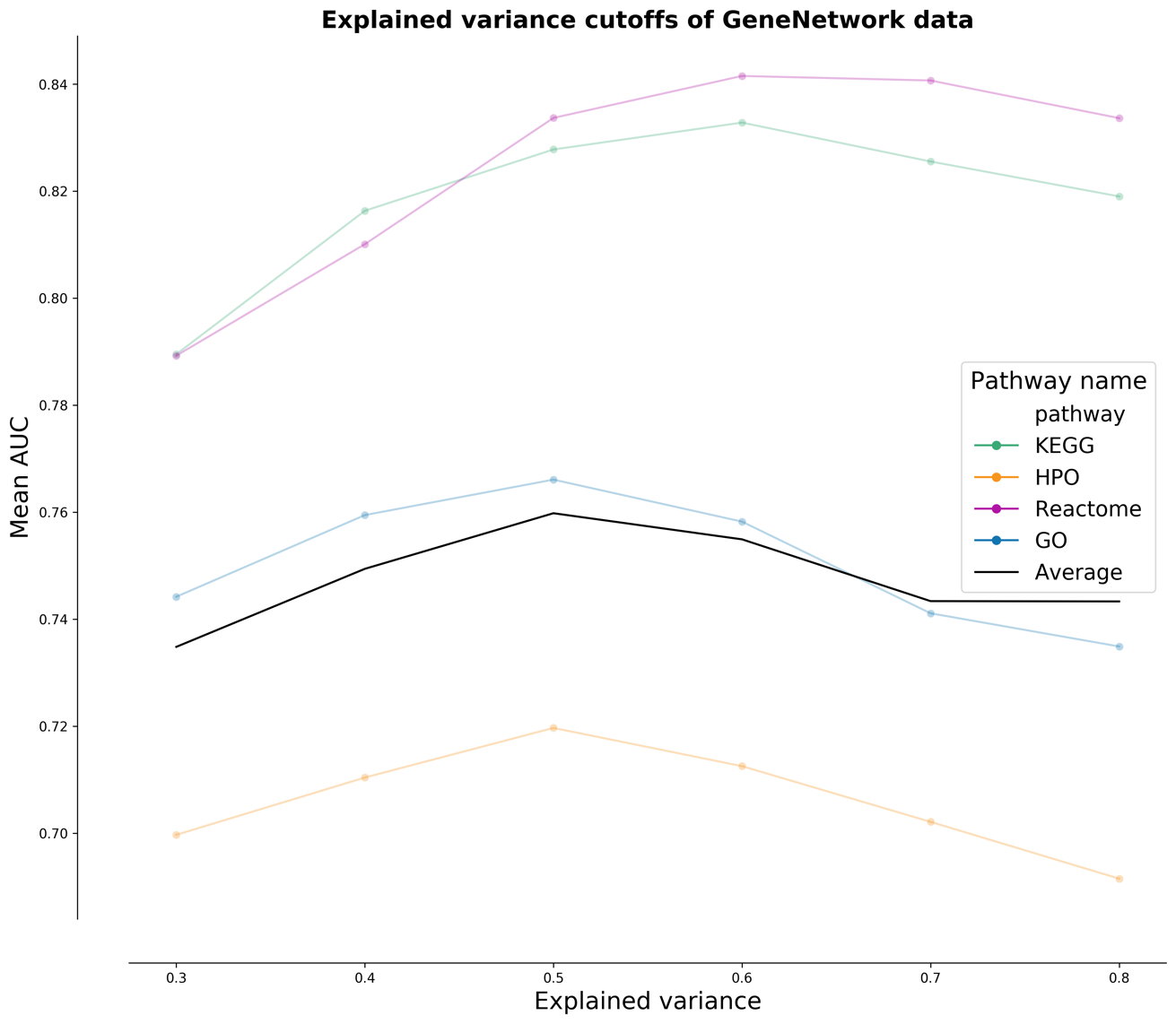


Figure S4: Selecting eigenvector cut-off value for kidney-derived samples data based on highest average AUC of different databases. An explained variance cut-off of 0.7 gives the highest prediction accuracy for the kidney-specific gene regulatory network based solely on 898 kidney-derived samples, averaged over several databases. Colored lines represent the mean AUC of the prediction of all pathways within one database. The black line represents the average AUC over all included databases. The number of eigenvectors included was based on the explained variance cut-off chosen.


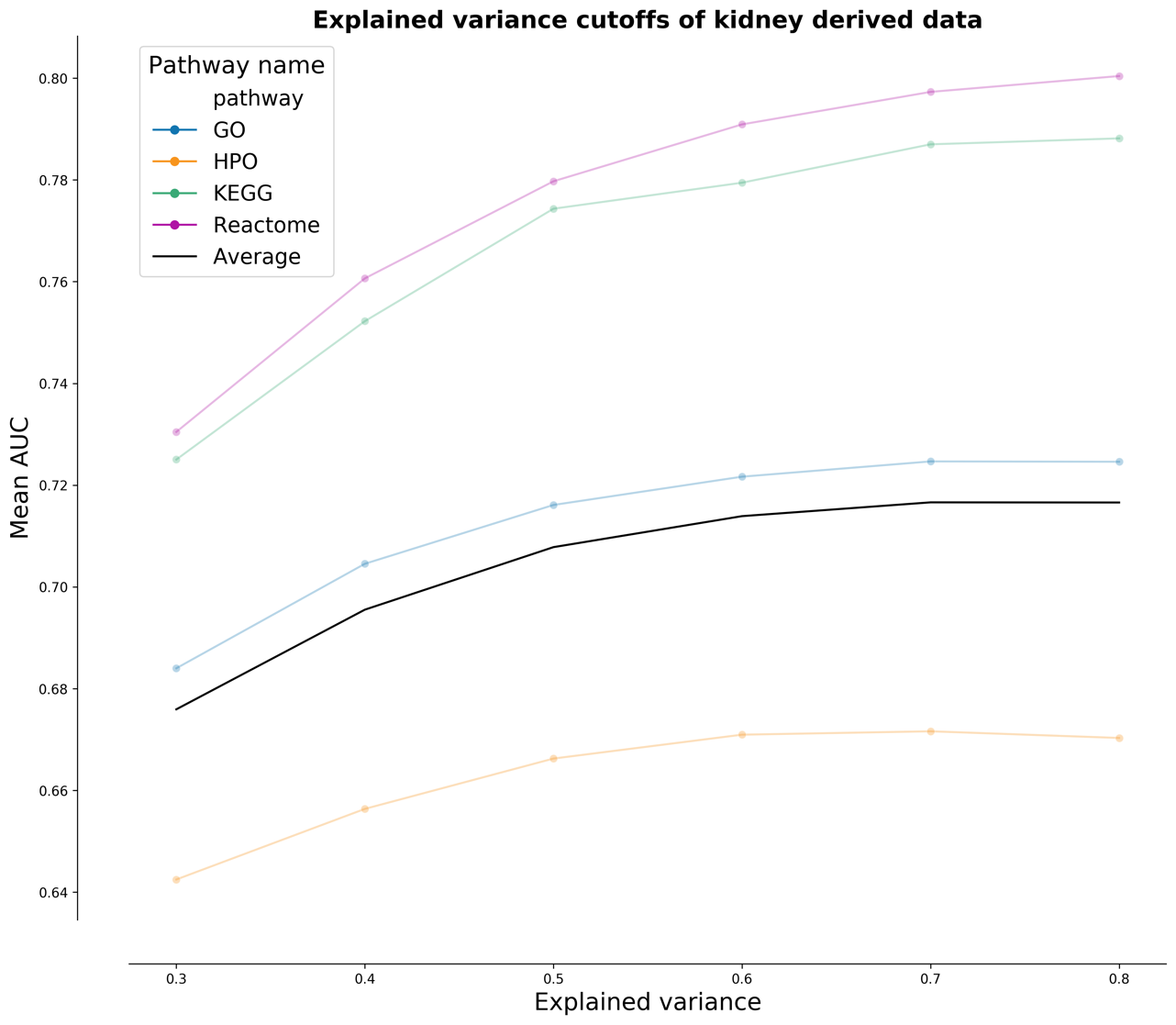


Figure S5: Pipeline of the updated network analysis. We start with a decomposition step of the input dataset to identify the components. These components are then used to fit a logistic model for each phenotype where the intercept and coefficients are used to calculate a gene log-odds score using the input components. The gene log-odds scores are translated to z-scores by permutation of the gene-components matrix to create a null distribution. This null distribution is used to calculate the gene log-odds scores of the null distribution, and the mean and standard deviation of these values are used to calculate the z-score for each gene.
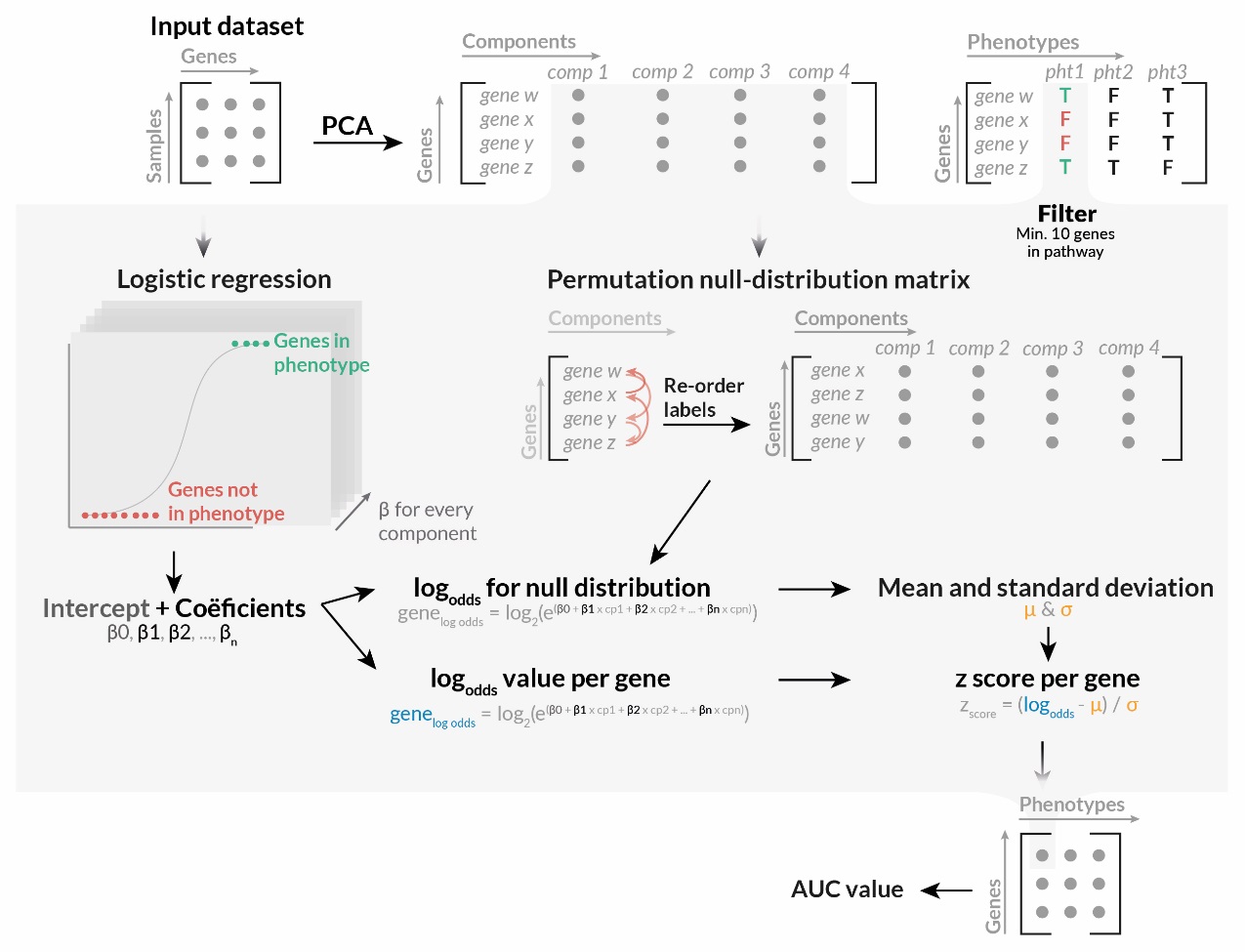


Figure S6: AUC value comparison between the original GeneNetwork and GeneNetwork based on the updated HPO database. The prediction accuracy of the original GeneNetwork pipeline described in the GADO paper (x-axis) compared to the same pipeline with the updated HPO database described in this paper (y-axis). Every dot represents the AUC value of one HPO-term. Color scale is based on the significance of the prediction in one, both, or neither method after multiple testing correction. The mean AUC of the original GeneNetwork is 0.69, and 1,751 pathways are predicted to be Bonferroni significant. The mean AUC of GeneNetwork with updated HPO data is 0.70, and 2,059 pathways are predicted to be Bonferroni significant.


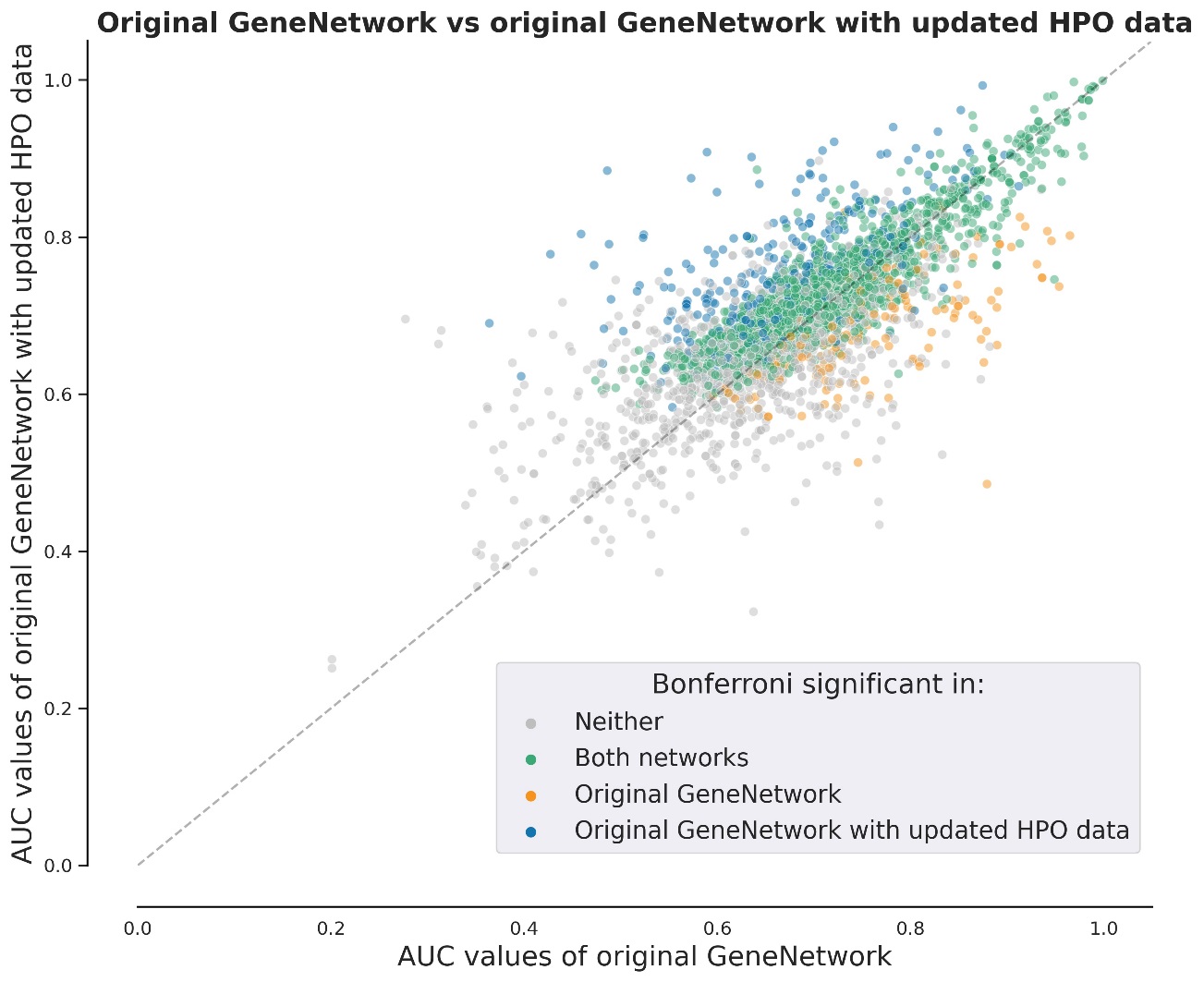


Figure S7: AUC value comparison between the original GeneNetwork pipeline and the updated GeneNetwork pipeline with the original HPO data. The prediction accuracy of the original GeneNetwork pipeline described in the GADO paper (x-axis) compared to the same pipeline with the updated HPO database described in this paper (y-axis). Every dot represents the AUC value of one HPO-term. Dot color is based on the significance of the prediction in one, both, or neither methods after multiple testing correction. The mean AUC of the Original GeneNetwork is 0.69, and 1,751 pathways are predicted to be Bonferroni significant. The mean AUC of the updated GeneNetwork with original HPO data is 0.71, and 1,911 pathways are predicted to be Bonferroni significant.


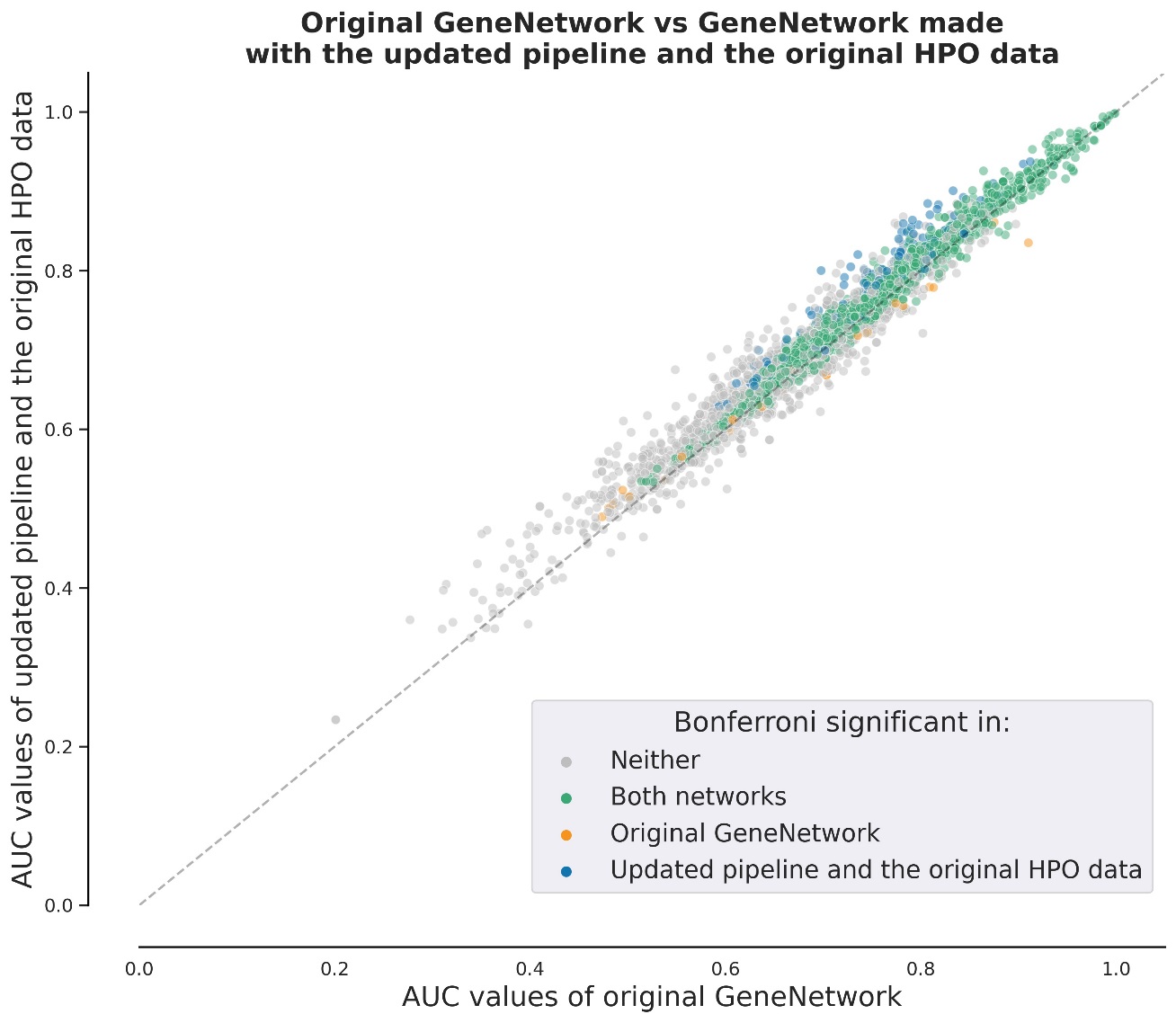


Figure S8: AUC value comparison between the original GeneNetwork and the updated GeneNetwork. The prediction accuracy of the original GeneNetwork pipeline described in the GADO paper (x-axis) is compared to the updated GeneNetwork (y-axis). Every dot represents the AUC value of one HPO-term. Dot color is based on the significance of the prediction in one, both, or neither method after multiple testing correction. The mean AUC of the Original GeneNetwork is 0.69, and 1,751 pathways are predicted to be Bonferroni significant. The mean AUC of the updated GeneNetwork is 0.72, and 2,266 pathways are predicted to be Bonferroni significant.


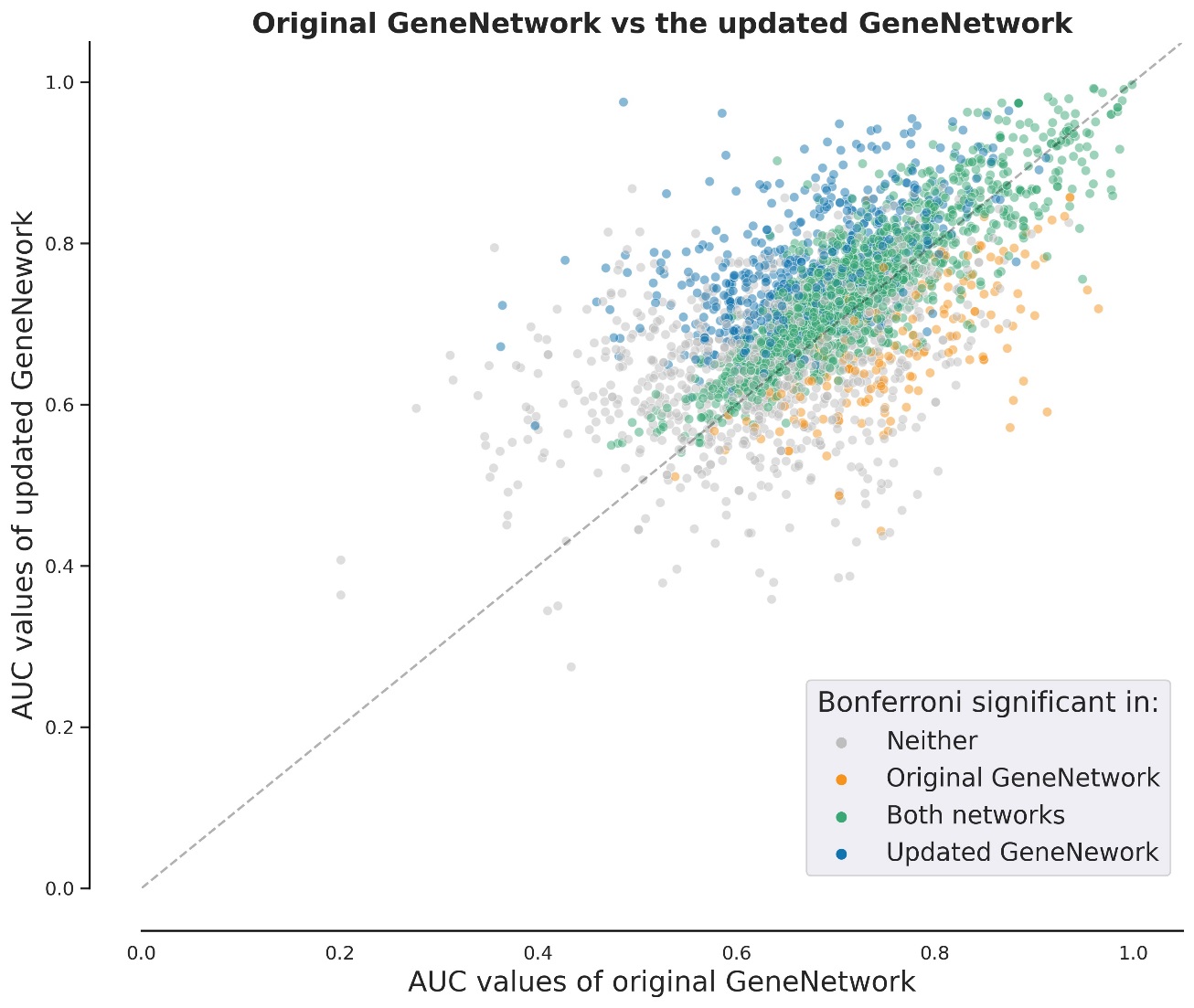


Figure S9: AUC value comparison of kidney-related HPO-terms between GeneNetwork and the network created with kidney-derived data. Kidney-related HPO-terms are predicted with a lower prediction accuracy compared to GeneNetwork. Furthermore, GeneNetwork is capable of predicting more kidney-related phenotypes compared to the network based solely on kidney samples.

Figure S10: ALG6-splice site analysis c.257+5G>A. RNA of the index patient LE1 was extracted from whole blood and submitted to RT-PCR. Obtained cDNA was subsequently amplified for *ALG6* exons 3-5. In the patient LE1, two bands one wild type (WT) and one lower band could be detected upon electrophoresis (A). The lower band was cut from the gel and subsequently submitted for direct sequencing. Chromatogram revealed an in-frame deletion of ALG6-exon 4 (p.del57_86) (B-C).


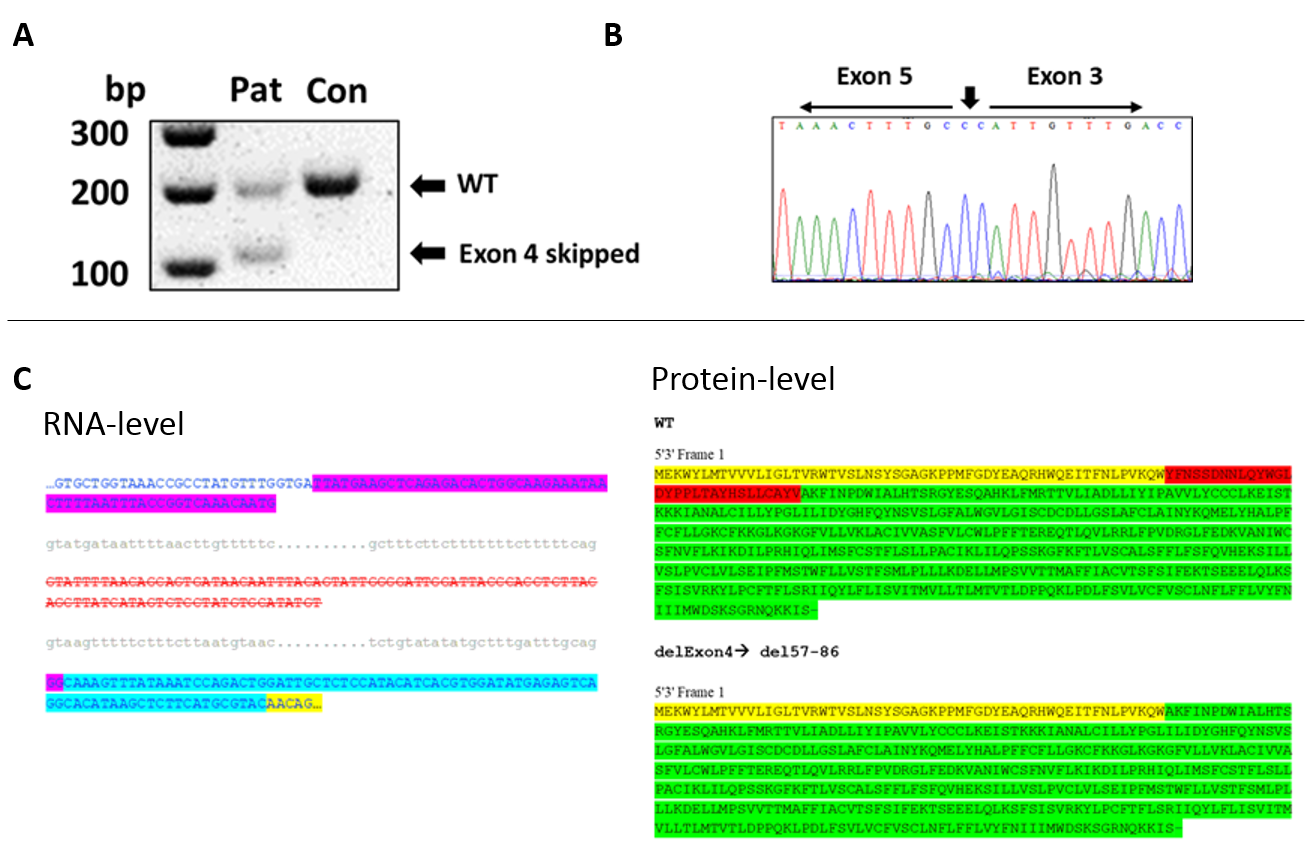


## Supplementary tables

### Table S1. Keywords for kidney sample selection

Keywords used for selection of kidney-derived RNA sequencing samples

*Supplied separately*

### Table S2. Sample annotations

Description of the kidney samples and the original studies.

*Supplied separately*

### Table S3. Kidney-specific HPO-terms

Overview of kidney-specific HPO-terms. Parent terms are shown, all child terms were included. We included HPO-terms specific to the kidney for benchmark analysis. We excluded HPO-terms related to kidney neoplasm and non-kidney specific child terms.

*Supplied separately*

### Table S4. Diagnostic kidney gene panel (n=379) of the University Medical Centre Utrecht NEF00v18.1

Genes that were checked for (likely) pathogenic variants that might be causing the phenotype of patients with an *ALG6* variant.

*Supplied separately*

### Table S5. Improved kidney-specific HPO-terms

List of improved kidney-specific HPO-terms in KidneyNetwork as compared to GeneNetwork.

*Supplied separately*

### Table S6. Phenotypes and genes in 13 kidney disease patients

Overview of phenotypes of the 13 patients that were analysed. For each patient, we prioritized genes using GADO with KidneyNetwork and intersected these with genes containing potentially pathogenic variants predicted by CAPICE. The resulting gene lists contained 1‒4 candidate genes for 9 of the 13 patients.

*Supplied separately*

## Supplementary notes

### Note S1. Kidney-specific RNA-sequencing samples

Meta-data of 3,108 publicly available human kidney-derived RNA-sequenced samples was downloaded from the European Nucleotide Archive (ENA) on October 1, 2019. In addition, 86 samples from the Genotype-Tissue Expression (GTEx) Project were obtained using dbGaP accession number phs000424.v8.p2.

### Note S2. Kidney-specific RNA-sequencing alignment

We used Kallisto^1^ to align the kidney expression dataset. The Kallisto index was based on Ensembl^2^ version 98 ncRNA and cDNA files (after removal of patch chromosomes) and made using default parameters, except for -k 31, and Kallisto version 0.43.1. Alignment was done using the Kallisto quant version 0.46.0 with default parameters and the addition of bootstrapping -b 30. For single-end data mapping, the additional parameters –l 200 and –s 20 were defined. The transcript counts were merged into gene counts. A TPM expression matrix was constructed based on the gene counts per sample.

### Note S3. Kidney specific sample and gene selection

We excluded samples with ≤ 70% mapping reads, samples sequenced on platforms other than the Illumina sequencing platform and peripheral blood samples. The expression matrix with the remaining samples was quantile-normalized and log_2_-transformed, followed by principal component analysis (PCA) over the samples. This method was first used for data originating from microarray RNA chips^3^ and later improved for RNA-seq data^4^. Based on the first two principal components (PCs), a cut-off for good quality samples was set at 0.030 **(Figure S1)**. We then excluded five of the samples that passed the PCA threshold but were sequenced using methods other than RNA-sequencing or ssRNA-sequencing, leaving 944 samples. Another 66 samples that correlated >0.9999 were removed because they were considered to be duplicates. The remaining 878 samples were used for further analysis **(Figure S2)**.

The reads were aligned to 59,562 coding and non-coding genes. We excluded 1,259 genes due to duplicate and/or no expression and 20 genes with zero variance, leaving 58,283 genes in the analysis.

### Note S4. Sample clustering and investigation using UMAP

UMAP values were generated using the umap() function in R version 3.5.1. We used PCs 1 and 2 as initial coordinates. Other parameters were defined as follows: n_threads = 24, n_epochs = 1000, n_neighbors = 100, min_dist = 0.1, init_sdev = 1e-4, learning_rate = 1, spread = 20, scale = "none" and nn_method = "fnn". For all other parameters, default values were used. We plotted the UMAP values using base R plot function. Samples are colored based on literature descriptions of the studies included **(Table S2)**.

Note S5. HPO filtering

We filtered the gene‒HPO-term annotations for “The molecular basis for the disorder is known; a mutation has been found in the gene” for OMIM and for “Modifying germline mutation(s)”, “Disease-causing germline mutation(s)”, “Disease-causing somatic mutation(s)”, “Disease-causing germline mutation(s) (loss of function)”, or “Disease-causing germline mutation(s) (gain of function)” for Orphanet. We then rebuilt the gene‒phenotype annotations in the HPO format that is used for network generation.

Note S6. Covariate identification

Covariate correction was done using Picard tools^5^. The covariate table was obtained using the command CollectRnaSeqMetrics with the following metrics: PCT_CODING_BASES, PCT_MRNA_BASES, PCT_INTRONIC_BASES, MEDIAN_3PRIME_BIAS, PCT_USABLE_BASES, INTERGENIC_BASES, INTRONIC_BASES, PCT_INTERGENIC_BASES and PCT_UTR_BASES. The option ‘strand_specificity’ was set to ‘first read transcription strand’ and the option ‘validation_stringency’ to ‘lenient’.

### Note S7. Decomposition

Gene expression was correlated using Pearson correlation and PC decomposition was performed over this correlation matrix. For this step, we used the PCA decomposition implemented by the Sklearn package^6^ version 0.22.2.post1 of the Python^7^ programming language version 3.6.3. The analysis was performed using the “full” svd_solver option.

### Note S8. PC threshold determination

Using explained variance cut-offs ranging between 0.3 and 0.8, the prediction accuracy (described in the next step) was calculated for pathways annotated in the HPO, Reactome^8^, KEGG^9^ and GO databases^10^ (Reactome, KEGG and GO databases downloaded on July 18, 2020). We chose to use the explained variance cut-offs that correspond to the highest average prediction accuracy, which were 0.5 for GeneNetwork and 0.7 for the network based only on kidney-derived data **(Fig. S3, Fig. S4)**. We selected the genes present in both datasets and then merged the eigenvector files.

### Note S9. Model fitting

Phenotypes with fewer than 10 annotated genes in the HPO database were excluded. For each phenotype, a logistic model was fitted between the genes annotated for that phenotype against all genes annotated to at least one other phenotype in the annotation file. If genes of the current phenotype were also included in other phenotypes, we excluded these genes from the second gene set to fit the model. The model was fitted using the LogisticRegression class of the sklearn package with the ‘lbfgs’ solver, L2 regularization, a C-value of 1.0, a tolerance of 1e-6 and a max iteration of 6000. The model resulted in an intercept (β_0_) and β values corresponding to every component (β_1_ until β_336_). We used these β values and the eigenvector scores to calculate a gene log-odds score for every gene in the eigenvector table with the following formula:

$${gene}_{log-odds-score}= \beta_{0} + \beta_{1}\cdot{eigenvector}_{1}+ \cdots+ \beta_{n}\cdot{eigenvector}_{n}$$

Where β_0_ is the intercept, β_1, …,_ β_n_ are the β values of the logistic model and the eigenvector_1, …,_ eigenvector_n_ are the eigenvectors values of a gene.

### Note S10. Leave-one-out cross validation

We fitted a new model for each annotated gene in which the gene‒phenotype annotation was set to false for that gene. This means that the newly trained model does not incorporate the gene as a known gene for that phenotype. We used the corrected intercept and β values to calculate the gene log-odds-score for the annotated genes.

### Note S11. Log-odds to z-score translation

We did a random imputation of the gene labels of the eigenvector matrix and calculated the gene log-odds-score using the same formula and β values from the already-fitted models. These null-distributed gene scores were used to calculate the average and standard deviation of the gene log-odds-score. We subsequently calculated the gene z-scores with the formula:

$${gene}_{z-score}= \frac{{gene}_{log-odds-score} - \mu}{\sigma}$$

Where μ is the average and σ is the standard deviation of the gene log-odds-scores of the null distribution.

We calculated the null-distributed gene log-odds-scores of the genes annotated to a phenotype by using the intercept and β values obtained from the models calculated during the overfitting step.

### Note S12. Patient selection and phenotype

The 13 patients included in the study are all suspected to have a monogenic kidney disease based on either family history, clinical presentation and/or early onset of their disease. However, no genetic cause had been found with diagnostic exome sequencing using trio-analyses or a diagnostic exome-based gene panel. Sequencing in these patients was performed at the University Medical Center Utrecht^11^. HPO-terms were assigned to these cases by two physicians from the genetics department.

### Note S13. (Manual) assessment of potential candidate genes

Variants were assessed based on gnomAD allele frequency, the ClinVar database, the HGMD database, Grantham score and prediction tools (CADD, SIFT, MutationTaster, PolyPhen-2, Splice Prediction Module in Alamut Visual v.2.15). Genes containing potentially harmful variants were assessed based on available literature about that gene or related genes and on known gene‒phenotype associations that could make this gene a more or less likely candidate. When autosomal recessive inheritance was expected based on the gene or the family history, and only one variant was found, the genetic data was reassessed for a second variant and SNP-array results -when present- were inspected for copy number variations. When sequencing data from parents was available, we assessed whether a variant was *de novo* or inherited.

### Note S14. Unsolved polycystic kidney and liver disease cohort

Patients with clinically or radiographically identified polycystic liver disease (PCLD) defined as >10 liver cysts, or autosomal dominant polycystic kidney disease (ADPKD) with no pathogenic mutation implicated in established disease genes underwent exome sequencing with variant calling analysis as previously described^14^. Rare variants with MAF <0.001 were assessed. For purposes of gene enrichment analysis, non-probands were excluded using VCFtools relatedness2 algorithm^15^, and genetically-defined ancestry was assessed using principle component analysis as previously described^16^.

### Note S15. 100,000 genomes project

Inclusion and genotyping of participants in the 100,000 Genomes Project, managed by Genomics England Limited (GEL), was previously described^13^. The multi-sample VCF dataset release v10, containing genome-wide sequencing data, was used to search for participants with a matching phenotype carrying rare variants in the candidate gene. We extracted high and moderate impact variants with a MAF < 0.001 and a scaled CADD-score (v1.5) of > 20 (or no CADD score if not applicable) and filtered for loss of function and known pathogenic variants. For all participants who remained with a variant in a candidate gene, we checked known kidney disease genes for (likely) pathogenic variants that might be causing their phenotype by checking whether a reportable variant was found in the diagnostic pipeline of the 100,000 Genomes Project and by checking for rare high/moderate impact variants in known renal genes defined by the complete diagnostic kidney gene panel of the University Medical Centre Utrecht NEF00v18.1 (Table S4).

# References

1. Bray, N. L., Pimentel, H., Melsted, P. & Pachter, L. Near-optimal probabilistic RNA-seq quantification. *Nat. Biotechnol.* **34**, 525–527 (2016).

2. Zerbino, D. R. *et al.* Ensembl 2018. *Nucleic Acids Res.* **46**, D754–D761 (2018).

3. Fehrmann, R. S. N. *et al.* Gene expression analysis identifies global gene dosage sensitivity in cancer. *Nat. Genet.* **47**, 115–125 (2015).

4. Deelen, P. *et al.* Improving the diagnostic yield of exome- sequencing by predicting gene–phenotype associations using large-scale gene expression analysis. *Nat. Commun.* **10**, 2837 (2019).

5. Picard Toolkit. (2019).

6. Pedregosa, F. *et al.* Scikit-learn: Machine Learning in Python. *J. Mach. Learn. Res.* **12**, 2825–2830 (2011).

7. Van Rossum, G. & Drake, F. L. *Python 3 Reference Manual*. (CreateSpace, 2009).

8. Jassal, B. *et al.* The reactome pathway knowledgebase. *Nucleic Acids Res.* **48**, D498–D503 (2020).

9. Kanehisa, M. & Goto, S. KEGG: kyoto encyclopedia of genes and genomes. *Nucleic Acids Res.* **28**, 27–30 (2000).

10. Ashburner, M. *et al.* Gene Ontology: tool for the unification of biology. *Nat. Genet.* **25**, 25–29 (2000).

11. Dubail, J. *et al.* SLC10A7 mutations cause a skeletal dysplasia with amelogenesis imperfecta mediated by GAG biosynthesis defects. *Nat. Commun.* **9**, 3087 (2018).

12. Sobreira, N., Schiettecatte, F., Valle, D. & Hamosh, A. GeneMatcher: A Matching Tool for Connecting Investigators with an Interest in the Same Gene. *Hum. Mutat.* **36**, 928–930 (2015).

13. Caulfield, M. *et al.* The National Genomics Research and Healthcare Knowledgebase. 4421856 Bytes (2019) doi:10.6084/M9.FIGSHARE.4530893.V5.

14. Besse, W. *et al.* ALG9 Mutation Carriers Develop Kidney and Liver Cysts. *J. Am. Soc. Nephrol. JASN* **30**, 2091–2102 (2019).

15. Danecek, P. *et al.* The variant call format and VCFtools. *Bioinformatics* **27**, 2156–2158 (2011).

16. Besse, W. *et al.* Isolated polycystic liver disease genes define effectors of polycystin-1 function. *J. Clin. Invest.* **127**, 1772–1785 (2017).
