# Supplementary material for: KidneyNetwork: using kidney-derived gene expression data to predict and prioritize novel genes involved in kidney disease
Source: Eur J Hum Genet. 2023 Feb 20;31(11):1300–8. doi: 10.1038/s41431-023-01296-x (PMC10620423; doi:10.1038/s41431-023-01296-x)

KidneyNetwork tutorial

This tutorial provides help in the navigation and use of the KidneyNetwork website: https://kidney.genenetwork.nl/. For more information about KidneyNetwork, please consult the preprint[1]. The application of KidneyNetwork used in the preprint is explained in paragraph 3.

How to obtain prediction scores per gene for a single phenotype?

1.1) To view the prediction scores for a pathway or phenotype from the REACTOME, HUMAN PHENOTYPE ONTOLOGY, KEGG or GO databases of interest, type the name of this phenotype in the search bar and hit <enter>.

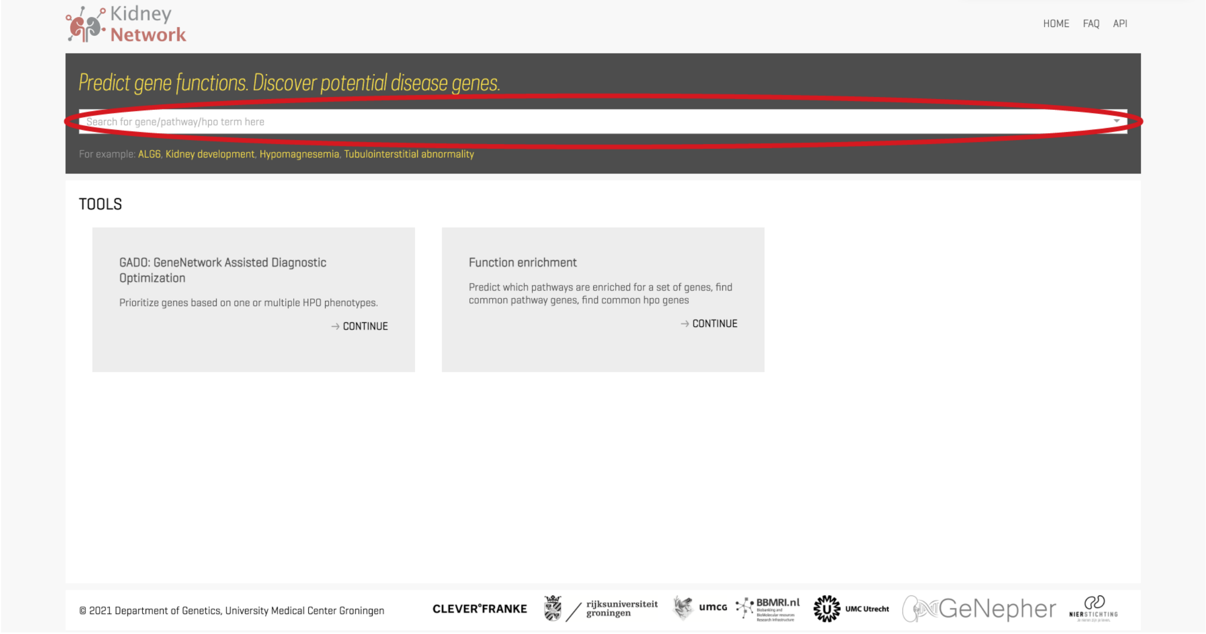

1.2) This will provide you with a page displaying a network of all predicted genes. Clicking on “PREDICTED GENES” will show the genes that this pathway or phenotype is predicted to be associated with. The genes are sorted for their prediction accuracy (p-value) and information about the correlation direction and the annotation status are provided. As genes have obtained a prediction score regardless of their annotation status, a gene predicted to be involved in a

pathway by KidneyNetwork can already be annotated for that same pathway.

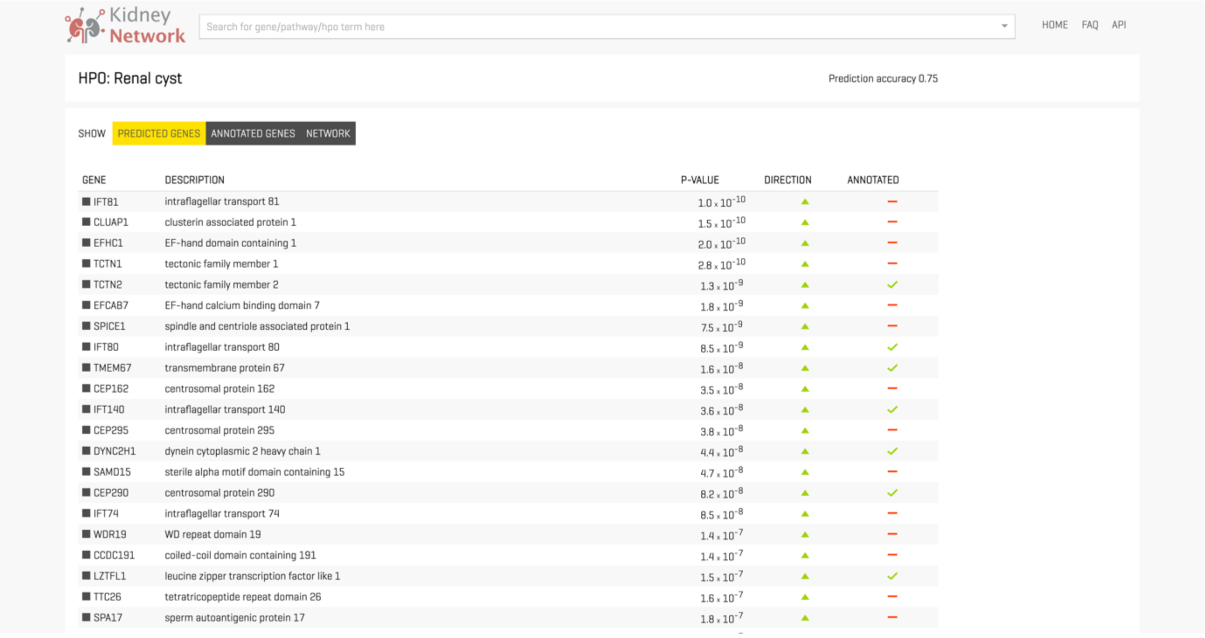

How to obtain prediction scores per phenotype for a single gene and optionally plot the co-expression networks of each predicted gene?

2.1) To view the prediction scores for a gene of interest, type the name of this gene name (HGNC symbol, entrez gene id) in the search bar and hit <enter>.

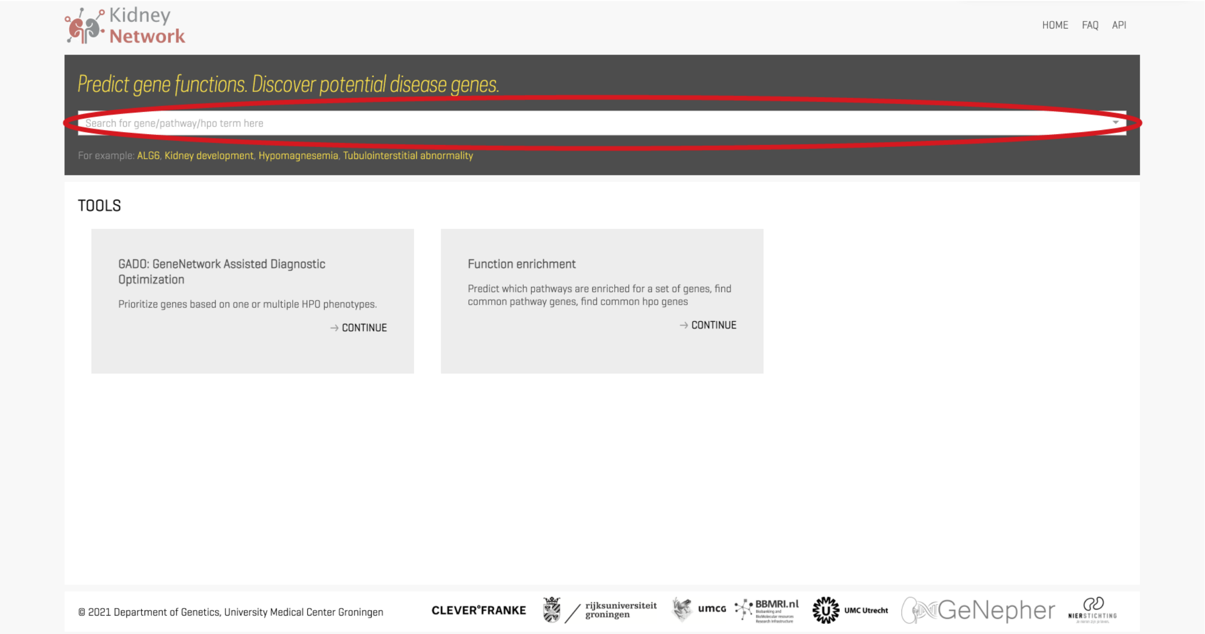

2.2) This will provide you with a page displaying the pathways and phenotypes that this gene is predicted to be associated with according to the REACTOME, HUMAN PHENOTYPE ONTOLOGY, KEGG and GO databases. The phenotypes are sorted for their prediction accuracy (p-value) and information about the correlation direction and the annotation status are provided. When clicking on a network symbol behind the term, the co-expression network containing all annotated

genes for this pathway or phenotype is shown.

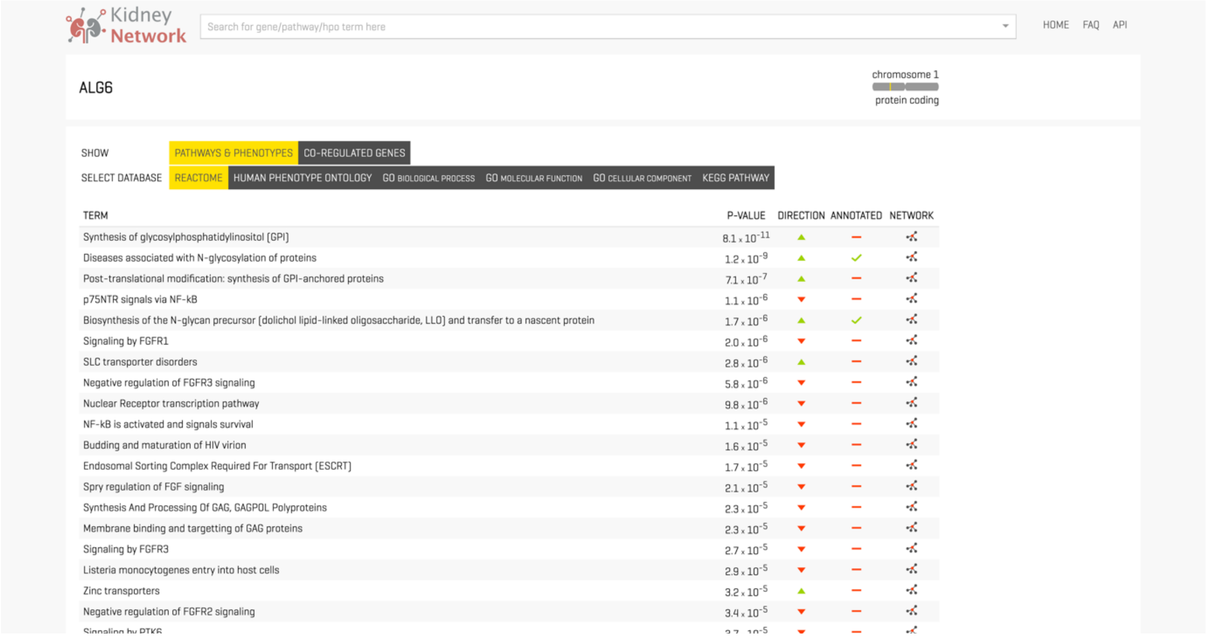

2.3) Edges are defined based on a z-score threshold of $z \geq 3$. This threshold can be adjusted using the threshold bar. The plot can be zoomed-in or -out using the + and - signs. This network can further visually be adjusted by clicking and dragging the genes. The network can be downloaded as a PDF file or a PNG file.

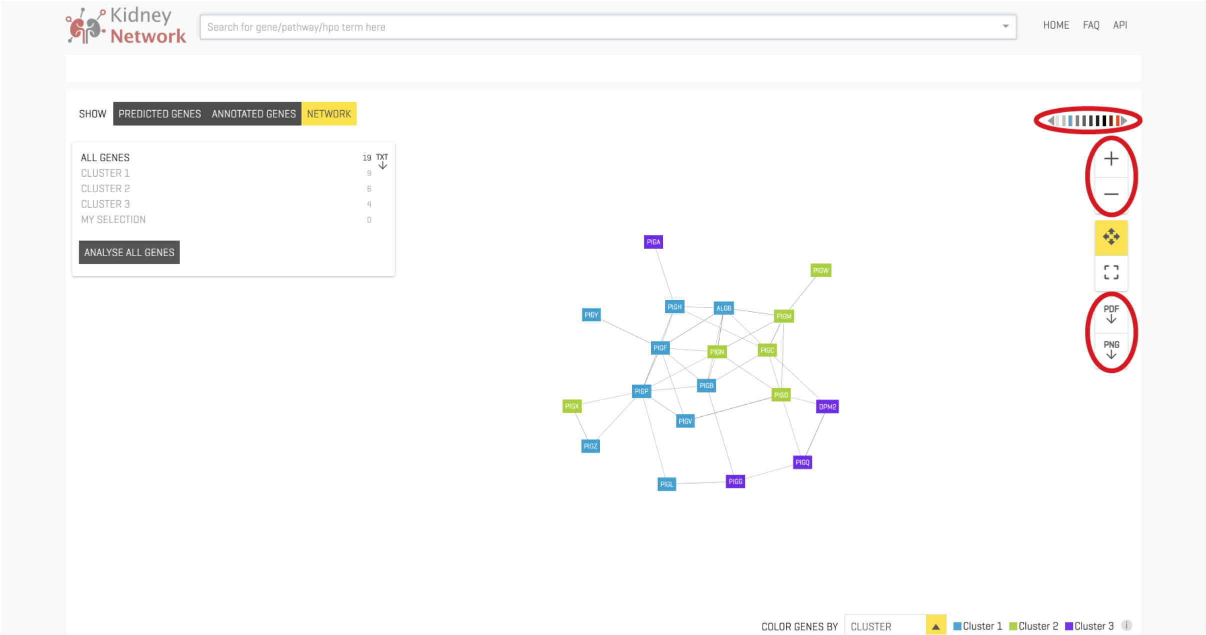

How to obtain the prediction scores per gene for a combination of phenotypes using the GADO algorithm?

3.1) The combined prediction scores of multiple pathways as calculated using GADO can be obtained by clicking on the GADO tool. Note: You will continue to see the KidneyNetwork name and logo in the upper left of screen indicating that this

algorithm is being applied to the optimized KidneyNetwork data.

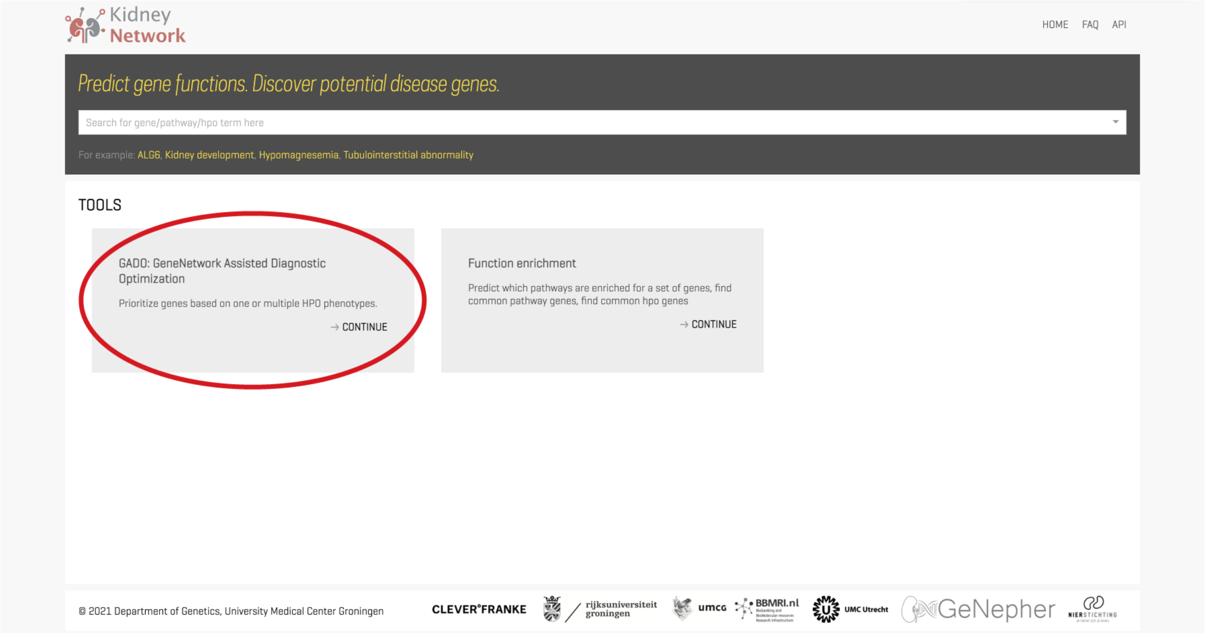

3.2) This will bring you to a screen where multiple HPO terms can be entered consecutively. For each entry, several HPO-terms are suggested to help with selecting the best match with the patient's phenotype. When selecting "OPTIONAL: filter output on candidate genes", a list of genes of interest can be pasted for filtering the results. If this option remains unchecked, prediction scores will be generated for all possible genes.

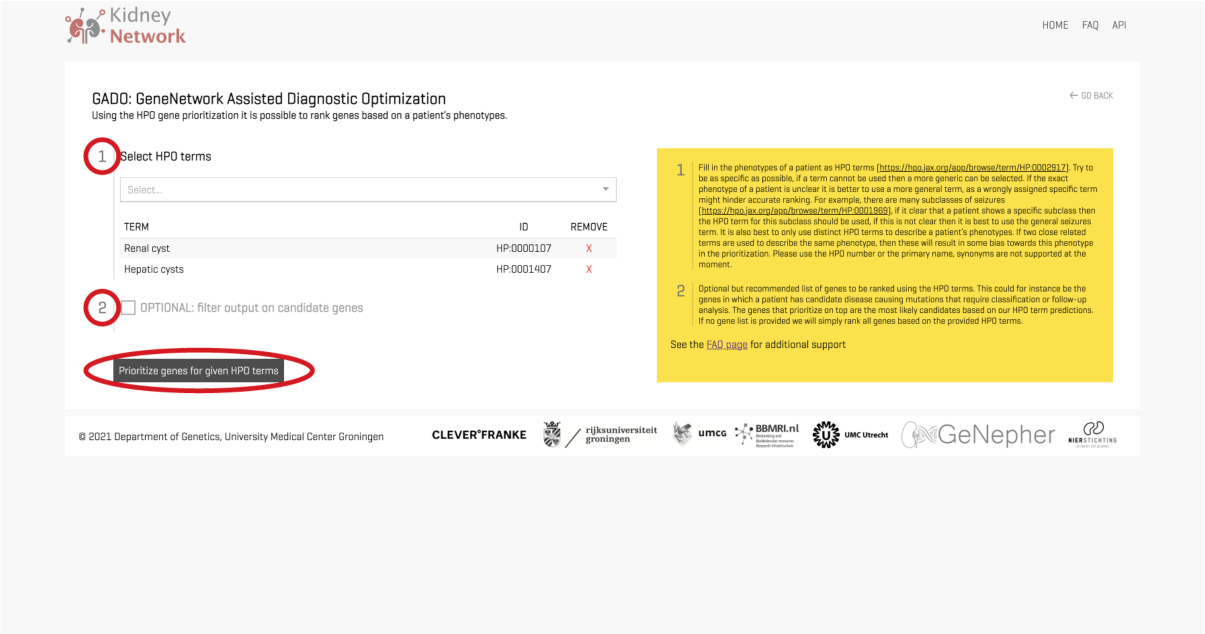

3.3) The results page will show the prioritized genes, ordered on their combined prioritization z-score. The z-scores of the individual terms are displayed as well. Furthermore, visual networks of each prioritized gene with its associated genes can

be obtained (see 1.3).

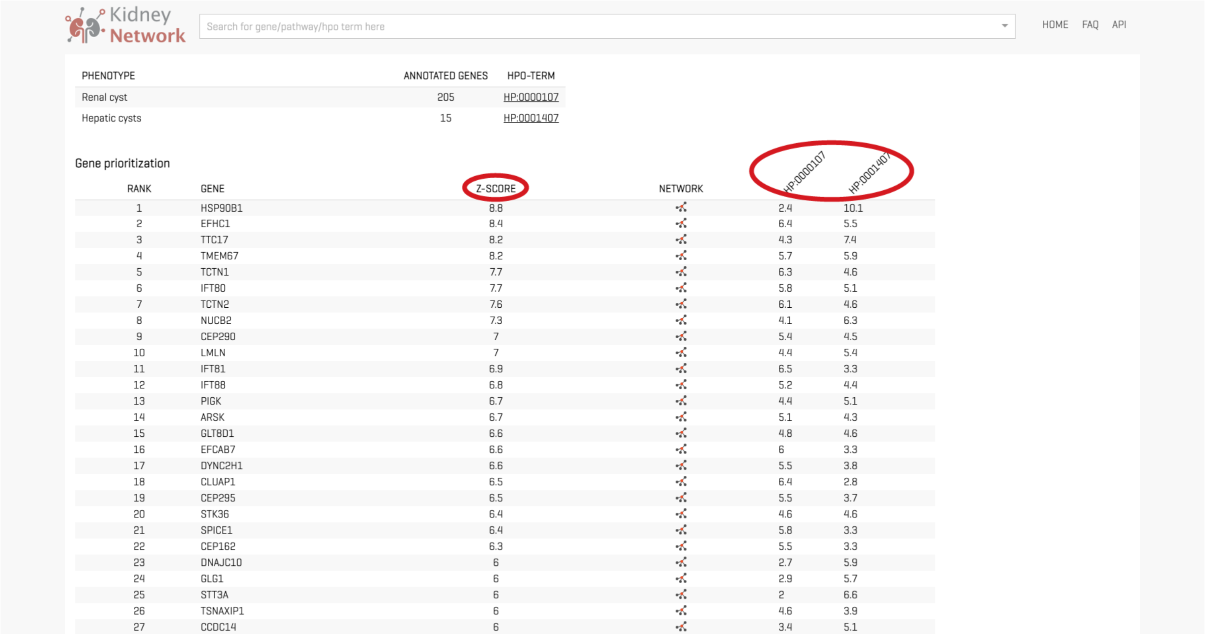

PHENOTYPE	ANNOTATED GENES	HPO-TERM
Renal cyst	205	HP:0000107
Hepatic cysts	15	HP:0001407

Gene prioritization

RANK	GENE	Z-SCORE	NETWORK	HP:0000107	HP:0001407
1	HSP90B1	8.8		2.4	10.1
2	EFHC1	8.4		6.4	5.5
3	TTC17	8.2		4.3	7.4
4	TMEM67	8.2		5.7	5.9
5	TCTN1	7.7		6.3	4.6
6	IFT80	7.7		5.8	5.1
7	TCTN2	7.6		6.1	4.6
8	NUCB2	7.3		4.1	6.3
9	CEP290	7		5.4	4.5
10	LMLN	7		4.4	5.4
11	IFT81	6.9		6.5	3.3
12	IFT88	6.8		5.2	4.4
13	PIGK	6.7		4.4	5.1
14	ARSK	6.7		5.1	4.3
15	GLT8D1	6.6		4.8	4.6
16	EFCAB7	6.6		6	3.3
17	DYNC2H1	6.6		5.5	3.8
18	CLUAP1	6.5		6.4	2.8
19	CEP295	6.5		5.5	3.7
20	STK36	6.4		4.6	4.6
21	SPICE1	6.4		5.8	3.3
22	CEP162	6.3		5.5	3.3
23	DNAJC10	6		2.7	5.9
24	GLG1	6		2.9	5.7
25	STT3A	6		2	6.6
26	TSNAXIP1	6		4.6	3.9
27	CCDC14	6		3.4	5.1

How to visualize the co-expression network of a custom set of genes?

4.1) Function enrichment of genes can be obtained by clicking on the "Function enrichment" tool. This will allow you to type or paste a list of genes or ensembl IDs. By subsequently hitting "open network", a visual network containing these genes will appear (see 1.3).

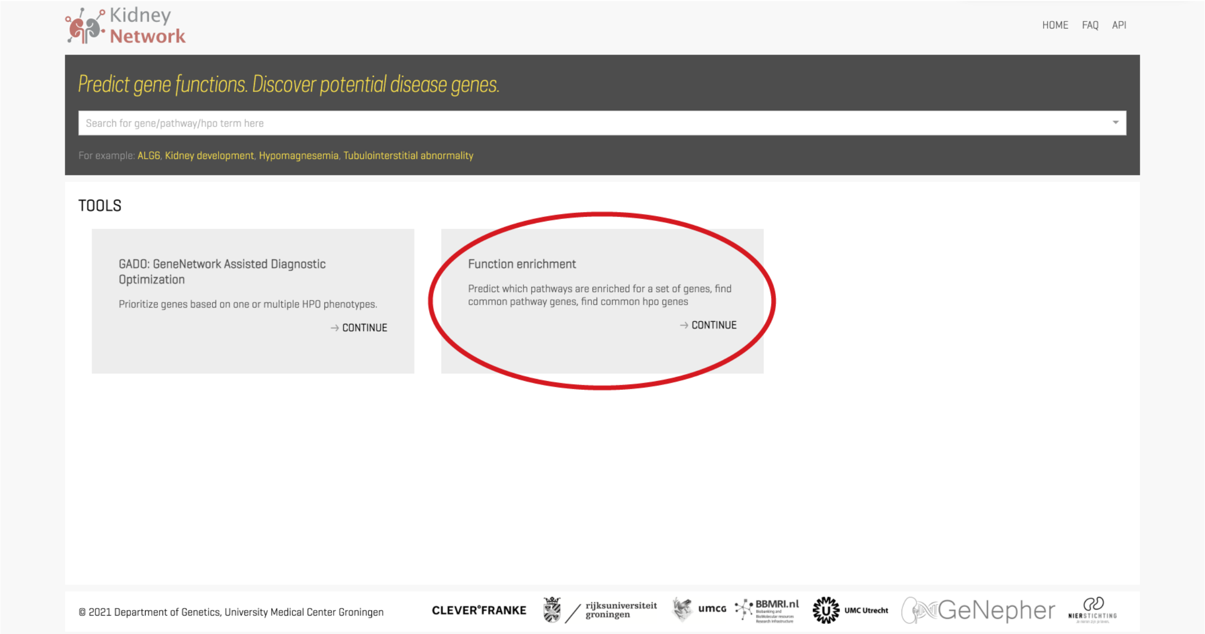

Supplement: Supplementary file 3 — KidneyNetwork website tutorial [file 41431_2023_1296_MOESM3_ESM.pdf]
